# Peroxidasin is essential for eye development in the mouse

**DOI:** 10.1093/hmg/ddu274

**Published:** 2014-06-03

**Authors:** Xiaohe Yan, Sibylle Sabrautzki, Marion Horsch, Helmut Fuchs, Valerie Gailus-Durner, Johannes Beckers, Martin Hrabě de Angelis, Jochen Graw

**Affiliations:** 1 Helmholtz Center Munich, German Research Center for Environmental Health, Institute of Developmental Genetics, Neuherberg, Germany,; 2 Institute of Experimental Genetics, Neuherberg, Germany,; 3 German Mouse Clinic, Neuherberg, Germany,; 4 German Center for Diabetes Research (DZD), Neuherberg, Germany and; 5 Chair of Experimental Genetics, Technische Universität München, Center of Life and Food Sciences, Freising-Weihenstephan, Germany

## Abstract

Mutations in *Peroxidasin* (*PXDN*) cause severe inherited eye disorders in humans, such as congenital cataract, corneal opacity and developmental glaucoma. The role of peroxidasin during eye development is poorly understood. Here, we describe the first *Pxdn* mouse mutant which was induced by ENU (*N*-ethyl-*N*-nitrosourea) and led to a recessive phenotype. Sequence analysis of cDNA revealed a T3816A mutation resulting in a premature stop codon (Cys1272X) in the peroxidase domain. This mutation causes severe anterior segment dysgenesis and microphthalmia resembling the manifestations in patients with *PXDN* mutations. The proliferation and differentiation of the lens is disrupted in association with aberrant expression of transcription factor genes (*Pax6* and *Foxe3*) in mutant eyes. Additionally, Pxdn is involved in the consolidation of the basement membrane and lens epithelium adhesion in the ocular lens. Lens material including γ-crystallin is extruded into the anterior and posterior chamber due to local loss of structural integrity of the lens capsule as a secondary damage to the anterior segment development leading to congenital ocular inflammation. Moreover, *Pxdn* mutants exhibited an early-onset glaucoma and progressive retinal dysgenesis. Transcriptome profiling revealed that peroxidasin affects the transcription of developmental and eye disease-related genes at early eye development. These findings suggest that peroxidasin is necessary for cell proliferation and differentiation and for basement membrane consolidation during eye development. Our studies provide pathogenic mechanisms of *PXDN* mutation-induced congenital eye diseases.

## INTRODUCTION

Anterior segment mesenchymal dysgenesis (ASMD; OMIM 107250) is a broad congenital manifestation of failures affecting the development of the cornea and lens of the eye. It is usually characterized by congenital corneal opacity, cataract, glaucoma, aniridia, eye coloboma and microphthalmia. The most common manifestation of ASMD with corneal opacity (around 80%) is Peters anomaly (OMIM 604229) and Axenfeld-Rieger syndrome (OMIM 180500) (1). We still know less about ASMD, although there are a number of mutations found in the affected patients. The majority of these mutated genes are transcription factors, such as *PAX6*, *FOXC1*, *PITX2*, *PITX3*, *FOXE3* and *AP2α* (2). These transcription factors were considered to regulate the mesenchymal cells to differentiate into distinct anterior segment tissues. Recently, there were a few other affected genes encoding extracellular matrix molecules, which are found to be related to anterior segment dysgenesis, such as *COL4A1* (3,4), *Col8a1* and *Col8a2* (5). These findings suggest a more complex molecular network regulating anterior segment development, as well as eye size and growth.

Recently, mutations in the human *PXDN* gene (OMIM 605158; encoding peroxidasin) were shown to cause a severe form of anterior segment dysgenesis, including corneal opacity, developmental glaucoma and congenital cataract (6). Since clinical symptoms in these patients are mainly present in the eyes, it is suggested that peroxidasin plays an important role during eye development. There are only a few reports dealing with the expression of *Pxdn* in the eye using model systems: in *Xenopus tropicalis*, peroxidasin was detected by *in situ* hybridization in the eye-forming region, especially in the developing lens (7). An *in situ* hybridization study in the mouse showed that peroxidasin is expressed in the lens cup and in the retina (8). However, the role of peroxidasin in eye development is unknown.

Peroxidasin is a conserved molecule combining multiple domains (leucine rich repeats, immunoglobulin domains and a von Willebrand factor domain) found in other extracellular matrix proteins and a peroxidase domain. Peroxidasin is up-regulated in p53-dependent apoptotic cells (9); it is also highly expressed in melanoma cell lines (10). Therefore, peroxidasin is also known as p53-responsive gene-2 or as Melanoma-associated gene-50 (MG50). Moreover, peroxidasin is highly expressed in the heart and vascular wall [therefore, it is referred to as vascular peroxidase 1 (VPO1; 11)].

Although the function of peroxidasin is still largely unknown, there are several studies showing that peroxidasin may play multiple roles in extracellular matrix formation, embryonic development, homeostasis and host defense. Peroxidasin was firstly identified in *Drosophila*; it is expressed by hemocytes and involved in the formation of the extracellular matrix (12). Furthermore, an *in vitro* study demonstrates that peroxidasin can be secreted from myofibroblasts and incorporated into the extracellular matrix; this process can be stimulated by TGFβ1 but does not seem to be mediated by the peroxidase enzyme activity (13). Further, disrupted extracellular matrix was also found in *C. elegans* with *peroxidasin* (*PXN*) mutations by electron microscopy. Additionally, mutations in *peroxidasin* also cause embryonic and larval lethality with variable epidermal phenotypes (14). Recently, it was shown that peroxidasin catalyzes the formation of the sulfilimine bonds (S=N) leading in *Drosophila* peroxidasin mutants to disorganized collagen IV networks and to turn visceral muscle basement membranes, pointing to a critical role for the enzyme in general tissue biogenesis (15). Peroxidasin also can generate hypochlorous acid, an antimicrobicidal agent, and may further play a role in host defense in human plasma (16). In addition, peroxidasin is discussed to be involved in endothelial cell apoptosis induced by oxidized low-density lipoprotein (17); it plays also a role in promoting oxidative stress, but the function of this extracellular matrix molecule is seemingly independent of its peroxidase activity in cardiovascular systems (18).

Besides the observation that mutations in the human *PXDN* genes lead to severe anterior segment ocular dysgenesis, the functional aspects of peroxidasin during eye development are largely unknown. Here, we report about the first *peroxidasin* mutation in the mouse; the mutant mouse was identified in course of an ENU-mutagenesis screen because of its severe ocular malformations. Therefore, our work provides novel insights into the role of *peroxidasin* during eye development, since its mutation in the mouse leads to a severe anterior segment dysgenesis including changes in cell proliferation and differentiation, basement membrane consolidation and regulation of inflammation.

## RESULTS

### Genetic analysis

**Figure 1.**
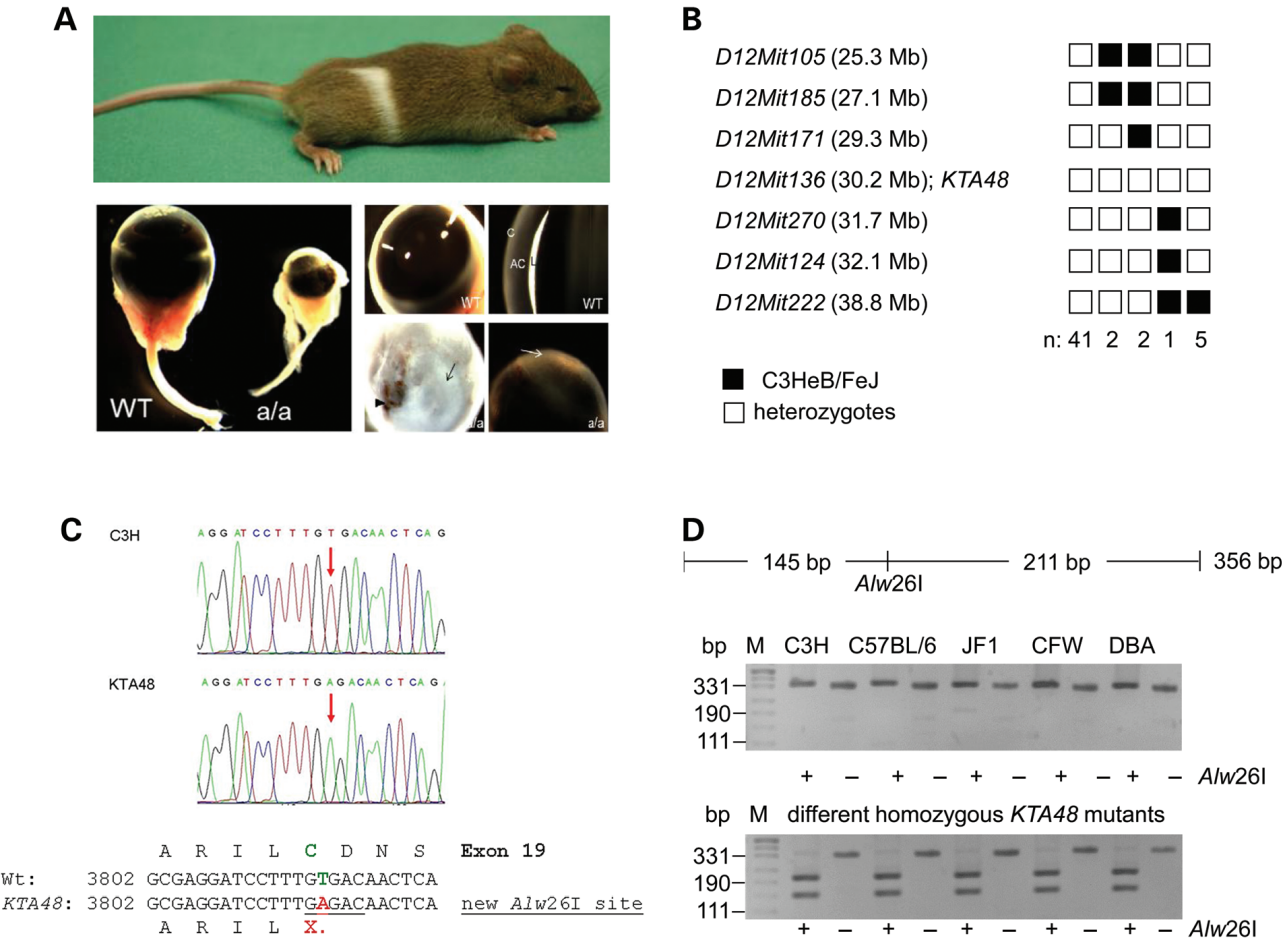
Genetics of the *KTA048* mutant mouse. (**A**) The recessive *KTA048* mutant was identified on C3HeB/FeJ background and showed a white spot at the belly and small eyes; the kinky tail is already lost. At adult (3 months), *KTA048* mutant eyes exhibit smaller eyes, corneal opacity and absent or very shallow anterior chamber. (**B**) Haplotype analysis revealed a critical interval of 2.4 Mb between the markers *D12Mit171* and *D12Mit270* including the marker *D12Mit136* and the candidate gene *Pxdn*. Black boxes illustrate the presence of two C3H marker alleles (recombination between microsatellite marker and *KTA048*); white boxes illustrate the presence of one copy of both alleles, C3H and B6 (lack of recombination). (**C**) Sequence analysis of the 3′ part of the *Pxdn* cDNA (PCR primer pair *Pxdn*-L5/R5; Table 1) revealed a T->C exchange at cDNA-position 3816 (red arrows) leading to a premature stop codon (X) in exon 19. The alignment of the DNA sequences together with the deduced amino acid sequences are given below; the new *Alw*26I restriction site in the mutant is underlined. (**D**) The *Pxdn*-mutation co-segregates within the mutant line. In the mutants, the *Alw*26I restriction enzyme cuts a genomic fragment of 356 bp into two fragments of 211 and 145 bp. DNA restriction analysis in different wild-type strains of mice showed the absence of T->A exchange. However, it is present in five homozygous (a/a) *KTA048* mutant mice randomly collected from the actual running breeding. The wild-type strains investigated are given above the gel; M: DNA size marker.

Offspring from ENU-treated male mice were screened for different phenotypic parameters including general dysmorphology (19,20). The mutant *KTA048* (kinky tail) was picked up because of its kinky tail and white spot at the belly; additionally, homozygous mutants showed microphthalmia and anterior segment dysgenesis including corneal opacity, severe iris bombé (forward-bowing iris) and very shallow or absent anterior chamber (Fig. 1A). During further breeding and selection for the small-eye phenotype, the kinky tail disappeared indicating that this phenotype is genetically not linked to the small-eye phenotype. However, the white spot at the belly is still co-segregating with the eye phenotype.

In a genome-wide linkage analysis using single nucleotide polymorphism (SNP) markers, the mutation was mapped to chromosome 12; markers within the interval between 30.0 and 32.4 MB showed a significant linkage to the mutated phenotype; the marker D12Mit136 (at pos. 30.9 Mb) did not show any recombination among 51 G3-mice tested (Fig. 1B). Based upon this positional information, we tested several candidate genes (*Sox11*, *Nrcam* and *Pxdn*), but no mutation was found in *Sox11* nor *Nrcam*. However, a mutation was identified in *Pxdn* cDNA (cDNA: T3816A in exon 19; Cys1272X; Fig. 1C), which causes a premature stop codon; the truncation is predicted to affect the C-terminal region of the peroxidase domain of Peroxidasin and the downstream von-Willebrand domain. The mutation creates also an additional recognition site for the *Alw*26I restriction enzyme, which can be used for genotyping. This mutation co-segregates within the breeding colony (5 mutant mice tested), but it does not represent a general polymorphism since it is not present in wild-type mice of different strains (C3H, B6, CFW, DBA/2J and JF1; Fig. 1D).

### Peroxidasin expression and eye development

**Figure 2.**
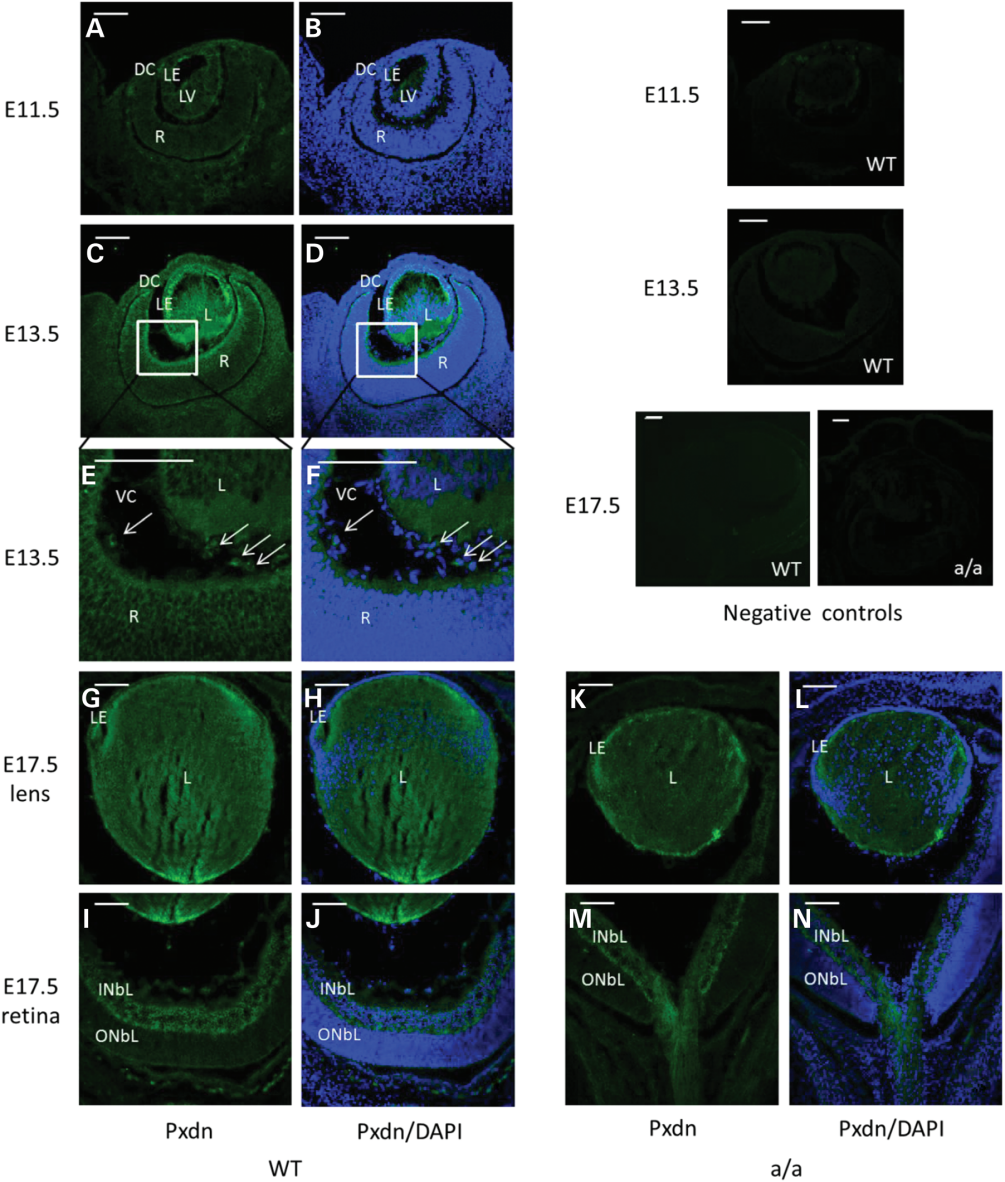
Peroxidasin during eye development. (**A** and **B**) Immunofluorescence studies showed weak Pxdn expression in the lens vesicle at E11.5. (**C**–**F**) At E13.5, it is highly expressed in the anterior lens epithelium, in the posterior lens fiber cells, in the inner limiting membrane (C and D) and in some mesenchymal cells in the vitreous (E and F, arrow); (E–F) are a close-up of (C) and (D). (**G**–**J**) At E17.5, Pxdn is mainly expressed in the lens epithelial cells, in the posterior part of the lens (G and H) and in inner neuroblastic layer (I and J). However, Pxdn is still expressed in the lens and in the inner neuroblastic layer and the expression pattern is similar to the wild-type eyes at E17.5 (**K**–**N**). Negative controls (without primary antibody) were shown in the most right panel. DC, developing cornea; L, lens; R, retina; LE, lens epithelium; LV, lens vesicle; INBL, inner neuroblast layer; ONBL, outer neuroblast layer; VC, vitreous cavity. Green: peroxidasin; blue: DAPI. Scale bar: 50 µm.

To understand the morphological consequences of the mutation, we first investigated the expression pattern of *Pxdn* during embryonic development using immunohistochemical studies (Fig. 2). At E11.5, the lens vesicle is just formed and peroxidasin is faintly expressed in the lens epithelium cells and in the anterior part of primary fiber cells (Fig. 2A and B). At E13.5, peroxidasin is strongly expressed in the developing lens especially the lens epithelium cells and in the inner limiting membrane (Fig. 2C and D). Additionally, it is also expressed in ocular mesenchymal cells in the vitreous (Fig. 2E and F). At E17.5, peroxidasin is not only expressed in the whole lens (Fig. 2G and H), but also in the inner neuroblast layer (Fig. 2I and J). In the lens, peroxidasin appears to be strongly expressed in the lens epithelial and at the posterior pole of the lens (Fig. 2G and H). In mutant eyes, Pxdn is still expressed in the lens and the inner neuroblast layer at E17.5, but the expression pattern is similar to wild-types (Fig. 2K–N).

**Figure 3.**
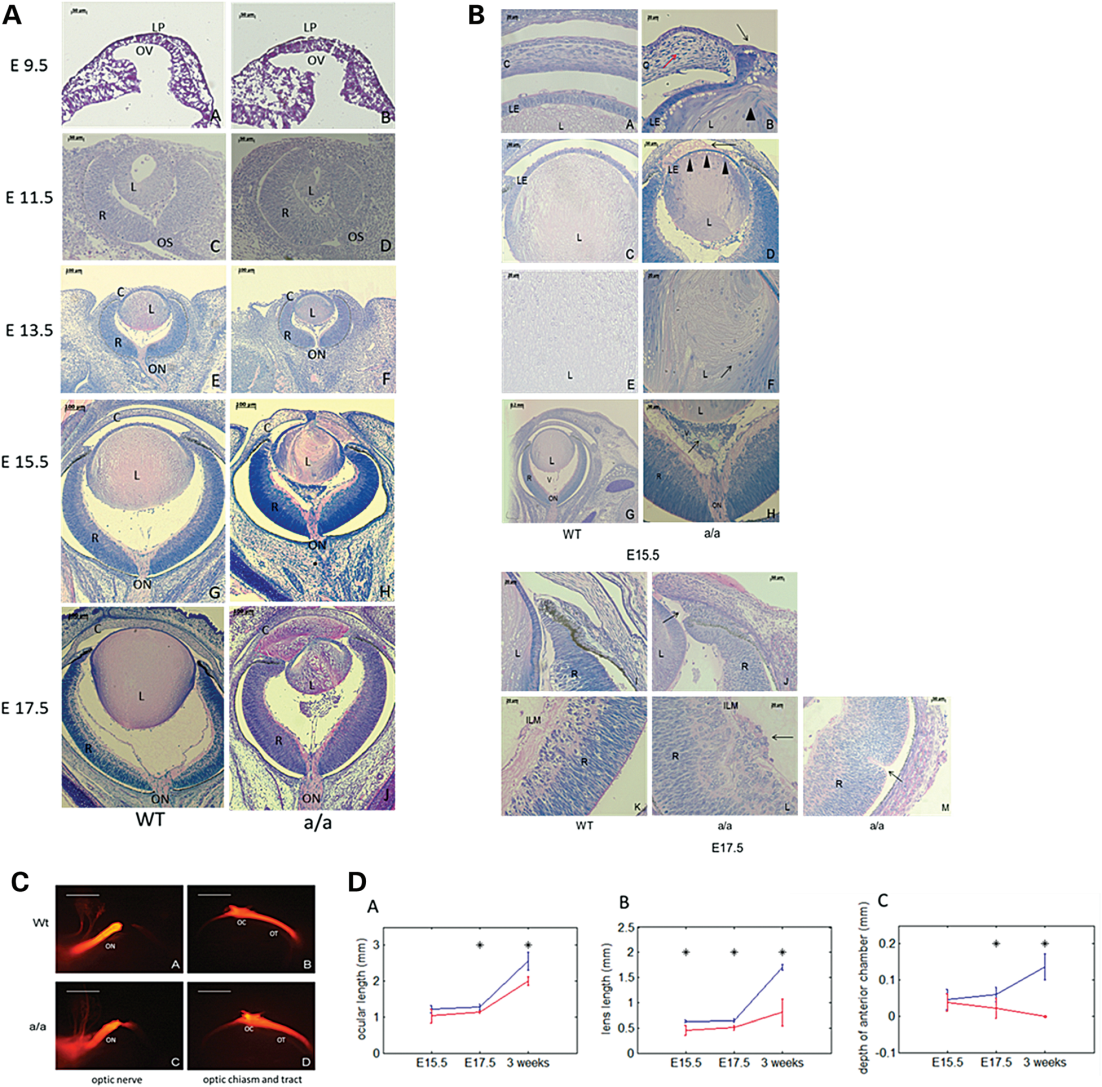
Anterior segment dysgenesis during eye development in *KTA048* mutants. (**A** and **B**) (A, a–h) At E15.5, the mutant eyes exhibit obvious anterior segment dysgenesis. The details are shown in (B, a–h): corneal-lens adhesion (b, black arrow), disorganized corneal stroma (b, red arrow), loss of structural integrity of the lens (b, arrowhead), lens fibers in the anterior chamber (b), thinner lens epithelium (d, arrowheads), disorganized lens matrix (f, arrow) and retrolental tissue (h, arrow). (A, i and j) At E17.5, the anterior chamber is further filled by lens material. Retinal phenotypes occur in a few cases (32.1%) including a loss of structural integrity in the ILM (B, l) and rosette-like structure (B, m). C, cornea; L, lens; LP, lens placode; OV, optic vesicle; R, retina; V, vitreous cavity; LE, lens epithelium; ON, optic nerve; ICA, corneal-iris angle; ILM, inner limiting membrane. Scale bar: (A) a–d, 50 µm; e–j, 100 µm. (B) a–b, e–f, i–m, 20 µm; c and d, h, 50 µm; g, 200 µm. (**C**) At E15.5, the crossing and fasciculation of the optic nerve, optic chiasm and optic tract are not affected in mutants (*N* = 5) (c and d) compared with wild-types (*N* = 5) (a and b). ON, optic nerve; OC, optic chiasm; OT, optic tract. Scale bar: 100 µm. (**D**) Impaired ocular and lens growth in *KTA048* mutants. (a–c) The ocular length, lens size and anterior chamber depth are significantly reduced in mutants compared with wild-types (*P* < 0.05). Blue line: wild-type; red line: mutants; asterisk: *P* < 0.05. Number: E15.5 (Wt 4, Mt 4); E17.5 (Wt 6, Mt 7); P21 (Wt 6, Mt 6).

Since *Pxdn* is expressed in the developing eye from E11.5 onward, we investigated eye development from E9.5 onward in homozygous *KTA048*-mutant embryos (the small-eye phenotype in the adult mice is recessively inherited). At E9.5 and E11.5, there are no obvious differences between wild-types and mutants (Fig. 3A, a–d). At E13.5, more mesenchymal cells are found in the vitreous of mutant eyes (Fig. 3A, e and f). At E15.5, the lens size of the mutants is significantly reduced compared with wild-type lens (*P* < 0.05), but the entire eye size is not yet affected at this stage (Fig. 3A, g, h and d). Moreover, the anterior segment is severely impaired in the mutant embryos compared with the wild-types (Fig. 3B, a–f). The anterior and posterior chamber including the iridocorneal angle is filled by lens fiber cells (Fig. 3A, h and b, d). Anterograde tracing was performed to investigate whether there are morphological differences in the optic chiasm and optic nerve between wild-types and mutants at E15.5, a stage when the optic chiasm has formed. No difference in the crossing and fasciculation of the optic chiasm and optic nerve was found between wild-types and *KTA048* mutants (Fig. 3C). At E17.5, the mutant eyes present a more severe ocular phenotype (Fig. 3A, j and b, j–m), and both the eye and lens size are significantly smaller than those in wild-types (*P* < 0.05). Moreover, the development of the anterior segment is further delayed in mutants; and the anterior chamber angle is filled by more lens fibers or mesenchymal cells (Fig. 3A, j and b, j).

At E15.5–E17.5, all mutants showed a loss of structural integrity of the capsule leading to extrusion of lens fiber cells into the anterior and posterior chambers (Fig. 3A, h, j; Fig. 3B, b, d; Table 4). In some cases (12/28, 42.9%), the lens vesicle remains attached to the cornea by a persistent lens stalk (Fig. 3B, b and Table 1). In 75% of the mutant embryos (21/28), mesenchymal cells aggregate and form the retrolental tissue in the vitreous (Fig. 3A, h and j; Fig. 3B, h; Table 1). The corneal stroma thickens, the parallel arrangement of the corneal stroma is distorted and the keratocytes are more densely distributed (Fig. 3B, b) and these corneal phenotypes are found in the mutants with persistent lens stalk. In most eyes, the retina develops well during embryonic development, although a few mutants (9/28, 32.1%) show a loss of structural integrity of the inner limiting membrane and retinal folds (Fig. 3B, l and m; Table 4).

**Table 1.** Summary of eye phenotypes in KTA048 homozygous mutants at E15.5–E17.5

Phenotype	Smaller lens	Disorganized lens matrix	Lens rupture^a^	Lens tissue in AC	Lens tissue out of the eye	Lens stalk	Mesenchymal cells in the vitreous	ILM defects and retinal folds
Number (28 eyes)	25/28	27/28	28/28	26/28	2/28	12/28	21/28	9/28
Percentage	89.3	96.4	100	92.9	7.1	42.9	75	32.1

These data are based on histological observation of embryonic eyes. AC, anterior chamber; ILM, inner limiting membrane.

^a^Short for the loss of structural identity of the lens capsule.

All mutant eyes and lenses analyzed were smaller than those in wild-type from E17.5 to adult (*P* < 0.05, Fig. 3D), and the depth of anterior chamber is progressively reduced from E17.5 to adult (*P* < 0.05, Fig. 3D). These quantitative data summarize that eye development and eye growth are severely affected in these mutant mice.

### Proliferation and lens differentiation

**Figure 4.**
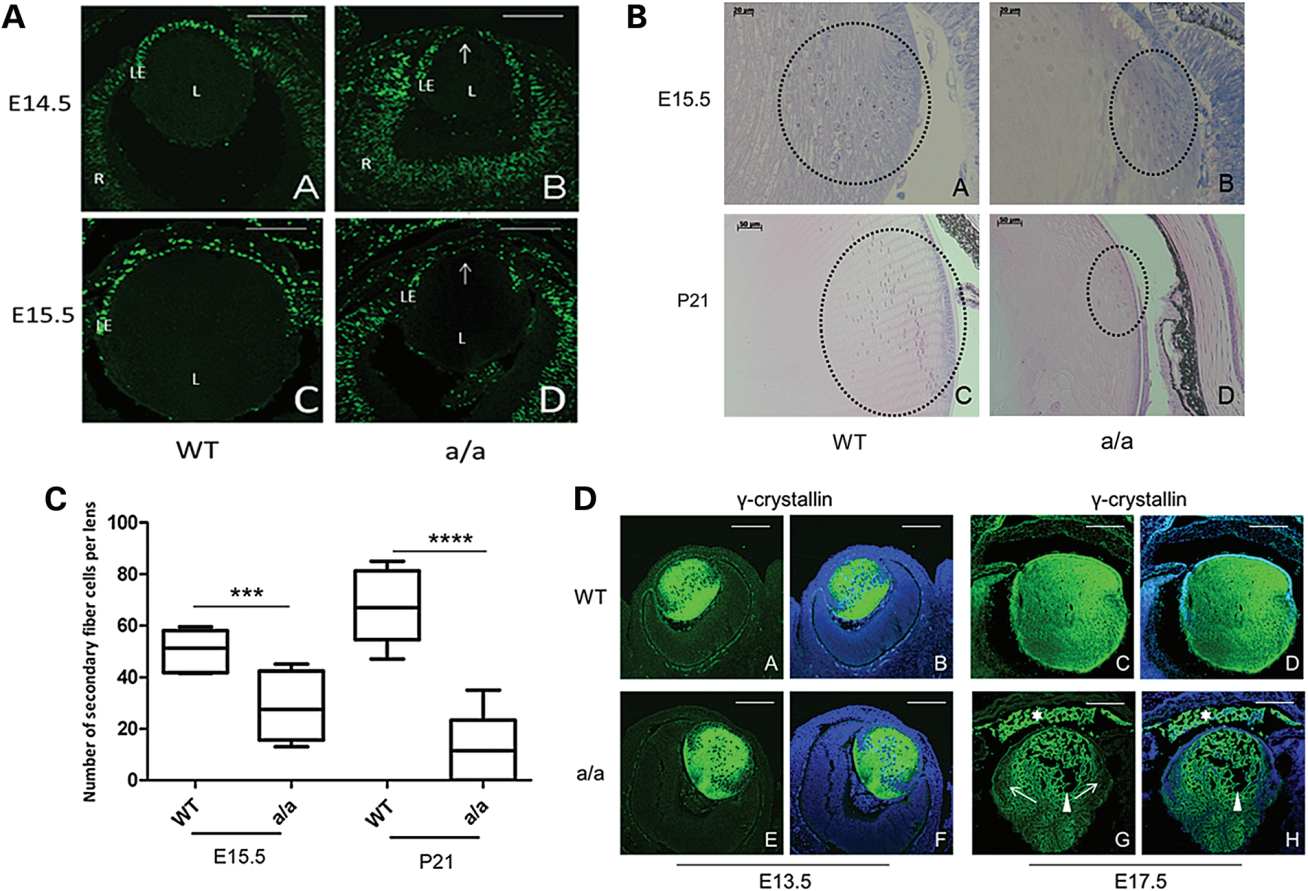
Reduced lens proliferation and disrupted lens differentiation in *KTA048* mutant embryos. (**A**) (a and b) At E14.5, wild-type lens epithelial cells (a) incorporated more BrdU than mutant lens cells in the central part of lens epithelium (b). (c and d) Similarly, also at E15.5, wild-type lens epithelial cells (c) incorporated more BrdU than mutant lens cells (d). L, lens; R, retina; LE, lens epithelium. The arrow points to the central epithelium. Scale bar: 50 µm. (**B** and **C**) The transition zone is disorganized and the number of secondary fiber cells is reduced in mutants (b) compared with wild-types (a) at E15.5 (C), ***P < 0.001. At P21, the epithelium cells in the transition zone are thinner and the number of fiber cells is obviously reduced in the mutants (d) compared with wild types (Cc, ****P < 0.0001). Scale bar: a and b, 20 µm; c and d, 50 µm. (**D**) (a–b and e–f) At E13.5, γ-crystallin is expressed in the whole wild-type lens (a and b); but there are no differences between wild-types (a and b) and mutants (e and f). (c–d, and g–h). However, the mutants showed reduced expression of γ-crystallin in the transition zone at E17.5 (g and h, arrow) compared with wild types (c and d), suggesting that the fiber cell differentiation is affected. Moreover, the expression of γ-crystallin was found in the anterior chamber of the mutants (g and h, white asterisk) accompanied by loss of lens fibers in the lens (g and h, arrowhead) due to the loss of structural integrity of the lens capsule and lens epithelium. Scale bar: a–h, 50 µm.

To investigate proliferation of the lens cells during eye development in *KTA048* mutants, pregnant mice at E14.5 and E15.5 were injected with BrdU and sacrificed 2 h later. At E14.5, BrdU labeling showed an intense cell proliferation of lens epithelium in wild-types, but a sparse distribution of BrdU-positive cells in the anterior lens epithelium of the mutants (Fig. 4A, a and b). At E15.5, there is a number of BrdU-positive cells in the anterior lens epithelium of wild-types, while very few positive cells are found in the anterior epithelium of the mutants, but the proliferation of the epithelial cells in the transition zone is not severely affected (Fig. 4A, c and d).

In addition to proliferation, lens differentiation is also affected in *KTA048* mutant eyes during embryonic development and postnatally. At E15.5, the nuclei of the transition zone, where the lens epithelia cells differentiate into the secondary lens fiber cells (21), are well organized as a bow-like pattern in wild-type lenses (Fig. 4B, a), whereas these cells in the mutants are disorganized (Fig. 4B, b) and the number of secondary fiber cells is significantly reduced in mutant lens compared with wild-type lenses (*P* < 0.01) (Fig. 4C). At P21, the defects in the transition zone are more severe: the number of secondary fibers in the transition zone is greatly reduced (*P* < 0.001) and the cells are disorganized (Figs 4B, c–d and 4C).

In line with the histology data, γ-crystallin as a marker for fiber cell differentiation (22,23) is highly expressed in the wild-type lenses (Fig. 4D, a and b) as well as in the mutant lenses (Fig. 4D, e and f) and no difference was found between wild-types and mutants at E13.5. However, γ-crystallin is ectopically found in the anterior chamber of the mutants (Fig. 4D, g and h) compared with wild-types (Fig. 4D, c and d) by immunofluorescence at E17.5, suggesting that lens crystallins leak out into the anterior chamber due to a loss of structural integrity of the lens capsule and lens epithelium. In addition, the expression of γ-crystallin is reduced in the secondary fibers at E17.5 in mutants (Fig. 4D, g and h) compared with the wild-types (Fig. 4D, c and d), suggesting that lens fiber cell differentiation is defect in *KTA048* mutants during embryonic eye development.

**Figure 5.**
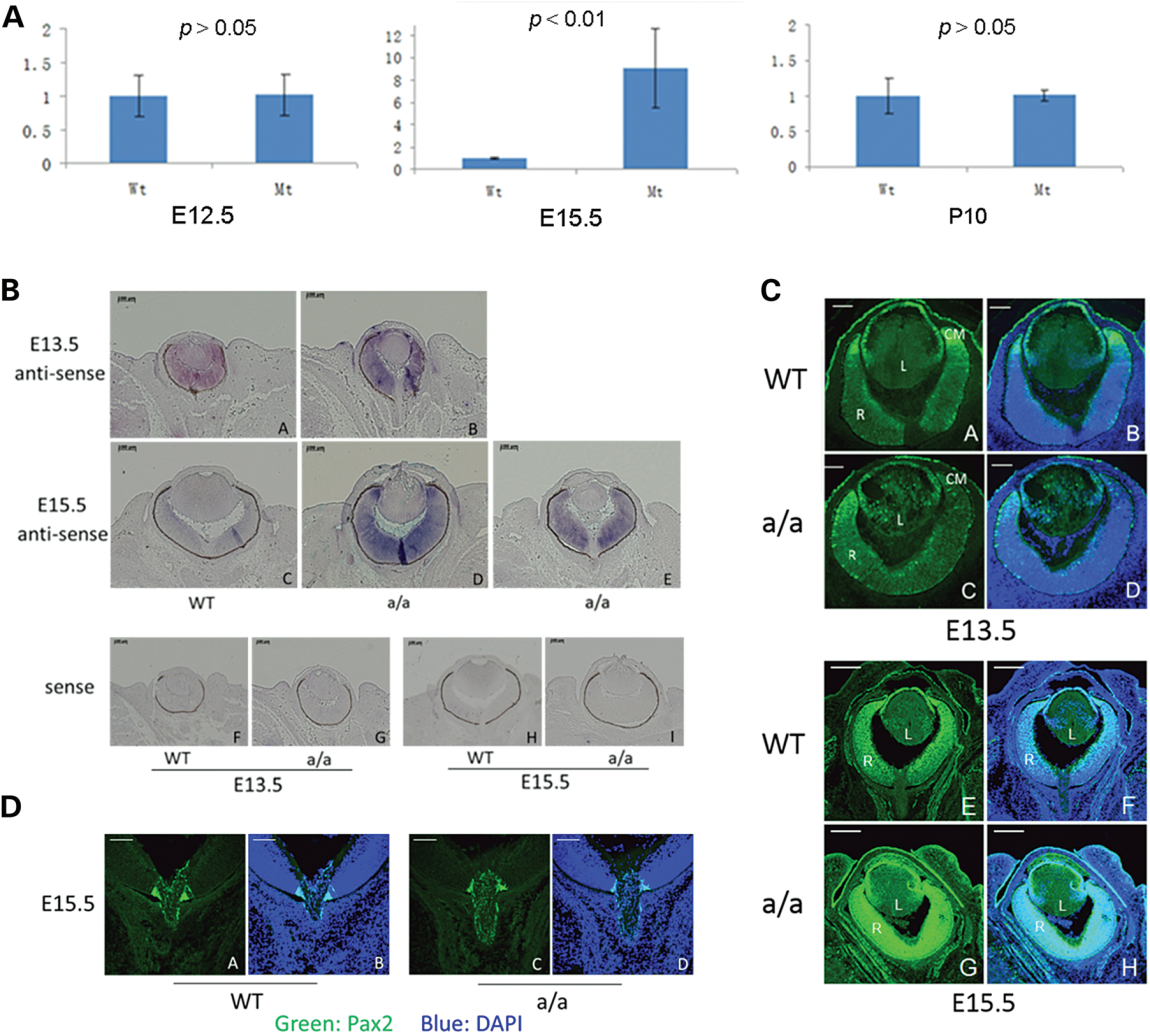
Pax6 expression pattern. (**A**) Real-time PCR showed that *Pax6* expression is up-regulated in mutant eyes compared with wild-type eyes at E15.5, although there is no difference of *Pax6* expression at E12.5 and P10. (**B** and **C**) At E13.5, *Pax6* mRNA is mainly expressed in the retina and lens epithelium [*in-situ* hybridization (B, a) and immunofluorescence (C, a, b)]. In the mutants, *Pax6* is not only expressed in the retina, but also expressed in the lens (A, b; C, c–d). At E15.5, *Pax6* is expressed in the lens epithelial cells and in the retina of wild-types (B, c; C, e–f). However, it appears that *Pax6* is up-regulated in the retina of the mutants (B, d–e). This result is also consistent with immunofluorescence data, demonstrating that the expression of Pax6 is stronger in the mutant eyes (C, g–h) at E15.5 compared with wild-types (C, e–f). (**D**) At E15.5, Pax2 is expressed in the optic nerve head especially in the junction between retina and the optic nerve head in wild-types (a and b). Compared with wild types, Pax2 expression is not affected in mutants (c and d). Scale bar: (B) a–i, 100 µm; (C) a–d, 50 µm; e–h, 200 µm; (D) a–d, 50 µm.

Using quantitative real-time PCR (qRT-PCR), we analyzed the expression of *Pax6*, which controls and regulates lens proliferation and differentiation. *Pax6*, a master control gene of eye development (24–26), is transiently up-regulated at E15.5 and then down-regulated to normal level at P10 in *KTA048* mutants (Fig. 5A). *In situ* hybridization and immunofluorescence were performed to confirm the qRT-PCR results. At E13.5, *Pax6* is highly expressed in lens epithelium and in the retina of wild-type eyes (Figs 5B, a and 5C, a–b). However, in the mutant eyes, *Pax6* is not only expressed in the lens epithelium, but also in the lens fiber cells, and the expression of *Pax6* appears to be decreased in the retinal ciliary margin in mutants (Figs 5B, b and 5C, c–d). The mRNA expression level of *Pax6* was not changed in mutants compared with wild-types at E12.5 as shown by qRT-PCR (Fig. 5A). At E15.5, *KTA048* mutants showed strong expression of *Pax6* in the eye, especially in retina (Fig. 5C, e–j), which was consistent with *in situ* hybridization data at E15.5 (Fig. 5B, c–e). However, the expression of Pax2 is not changed in mutants at E15.5 compared with wild-types (Fig. 5D, a–d), indicating that the optic nerve development is not affected at E15.5, when the anterior segment dysgenesis occurs.

**Figure 6.**
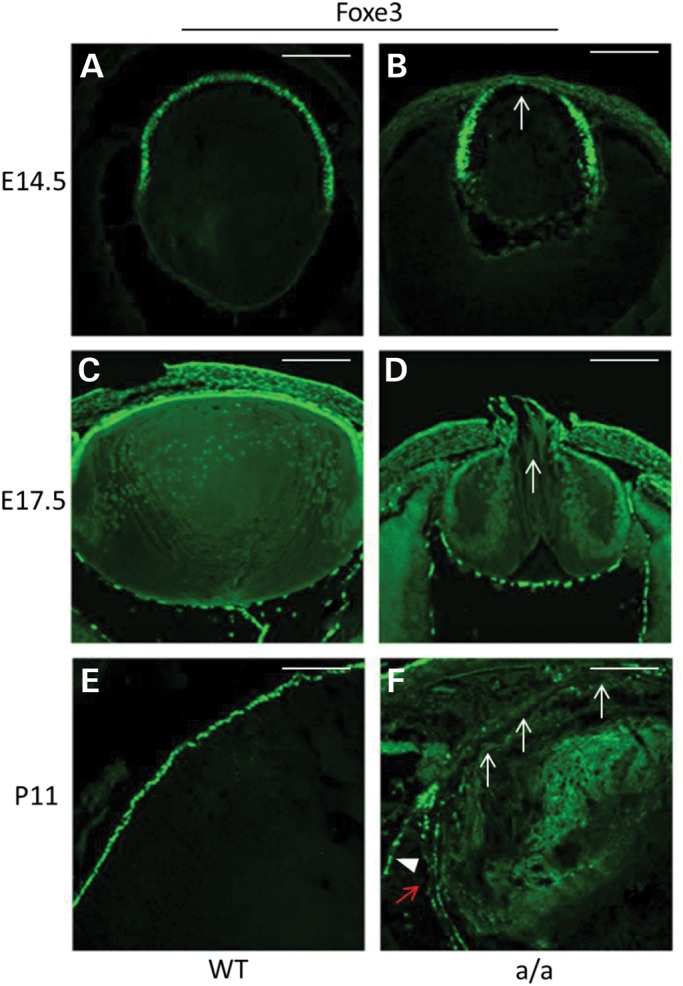
Aberrant expression of Foxe3 in mutants. (**A**–**F**) At E14.5, there is robust expression of Foxe3 in lens epithelial cells in wild-types (A), whereas in the anterior lens epithelium of the mutants the expression of Foxe3 is decreased (B, arrow). At E17.5, there is still strong expression of Foxe3 in lens epithelia cells in wild-types (C), but decreased expression of Foxe3 in mutants (D, arrow). At a later stage (P11), Foxe3 is only expressed in lens epithelium cells (E) while in mutants Foxe3 expression is completely absent in most lens epithelium cells (F, white arrow) and decreased expression of the other lens epithelium cells (F, red arrow) and it is ectopically expressed in some regions outside of the lens (F, arrowhead). Scale bar: 50 µm.

Foxe3 is necessary for lens proliferation and differentiation (21,27–29). Staining for Foxe3 was performed in the eyes at E14.5, E17.5 and P11. In the wild-type lens, Foxe3 is robustly expressed in the lens epithelial cells at different stages (Fig. 6A, C and E). In the mutant lens, we observed at E14.5 and E17.5 a reduced expression of Foxe3 in the central part of the lens epithelium, which is also the region incorporating less BrdU (Fig. 6B and D). At P11, the expression of Foxe3 is greatly reduced and some regions showed no expression of Foxe3, and Foxe3 expression can be found in the cells outside of the lens, suggesting that the lens epithelial cells are severely disrupted in the postnatal period (Fig. 6F).

### Ocular inflammation in *KTA048* mutant mice

**Figure 7.**
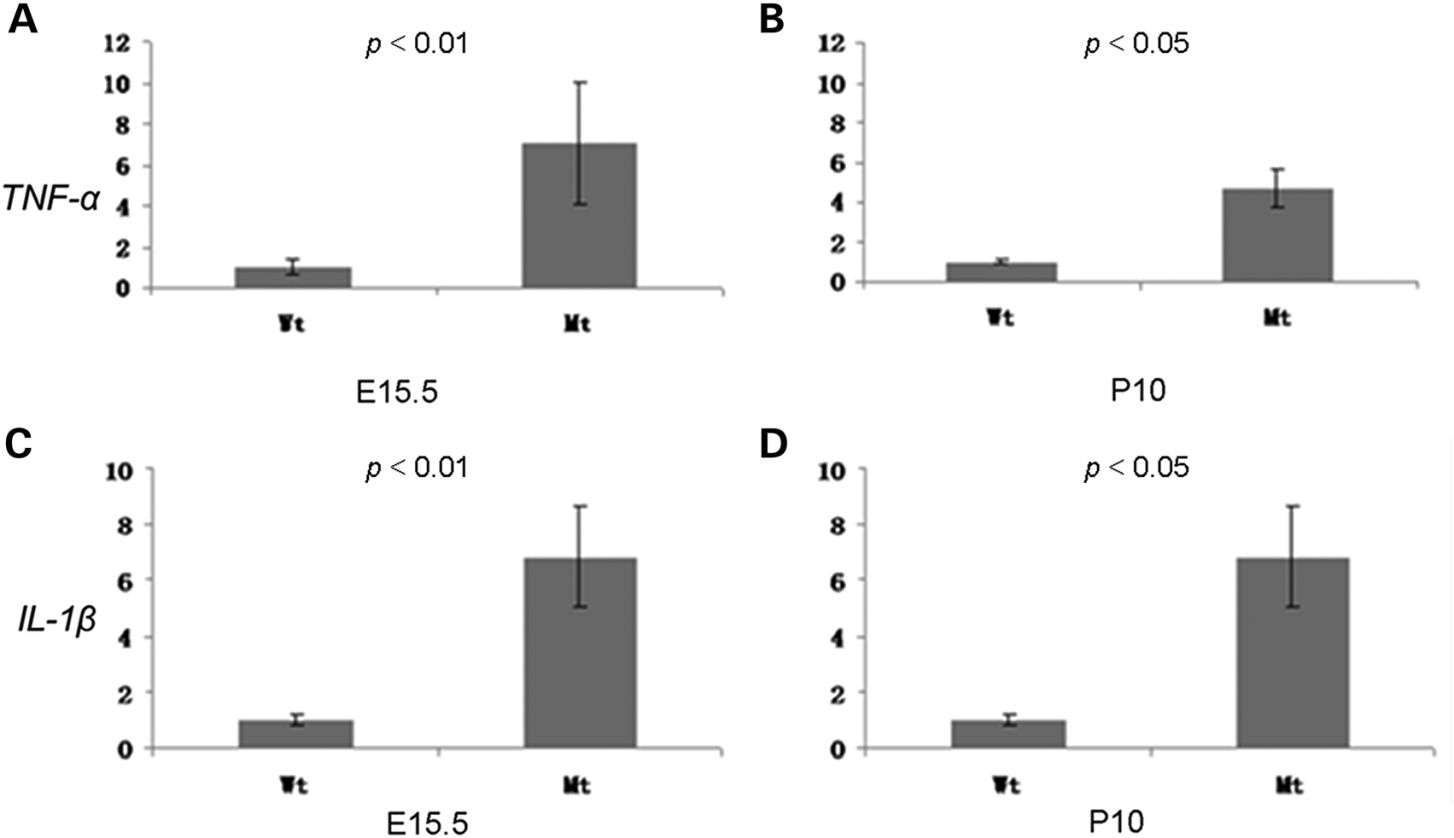
Ocular inflammation during eye development. Real-time PCR showed that the expression of inflammation marker genes (*TNF-α* and *IL-1β*) are significantly up-regulated in mutant eyes at E15.5 and P10, which suggests that ocular inflammation occurs during embryonic eye development and continues at postnatal period. Mt: a/a; E15.5 (n(Wt) = 4; n(Mt) = 6); P10 (n(Wt) = 4; n (Mt) = 4).

The poor anterior segment development in conjunction with the presence of crystallins outside of the lens may increase ocular inflammation during eye development, which might be also strengthened because of the loss of the peroxidase function of the mutated *Pxdn* gene. Therefore, we compared quantitatively the expression of different genes which are critical for inflammation (*TNFa* and *IL-1b*) in the eyes of *KTA048* mutants and wild-types at E12.5, E15.5 and P10 by qRT-PCR. The expression of *TNF-α* and *IL-1β* is up-regulated at E15.5 in mutants (*P* < 0.01) and continues to be up-regulated at P10 (*P* < 0.05) (around 4 days before eyes open in mice; Fig. 7A–D). These data suggest that congenital ocular inflammation occurs in the *KTA048* mutants.

### Anterior segment dysgenesis in *KTA048* mutant mice at postnatal periods

**Figure 8.**
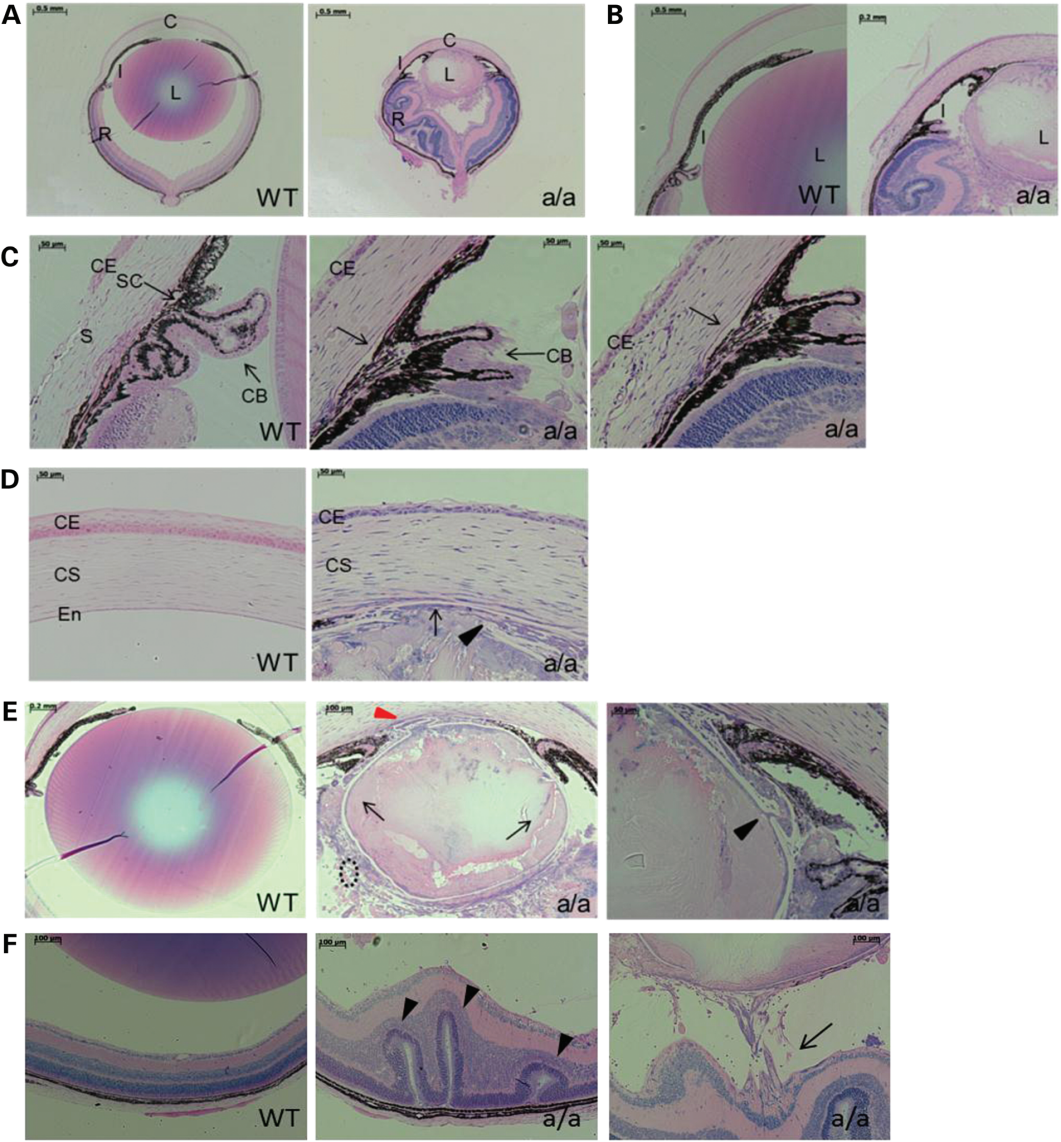
Anterior segment dysgenesis in *KTA048* mutants at the age of 3 weeks. At P21, the mutants displayed remarkable smaller eyes and lenses compared with wild-types (**A**) and severely anterior segment dysgenesis including iris hypoplasia and underdevelopment of ciliary body (**B** and **C**), lens-corneal adhesion (**D** and **E**), thinner corneal epithelium and disorganized corneal stromal keratocytes (D), disorganized lens matrix which leads to congenital cataract (E). The iris is attached to the cornea and lens (A, B and E), resulting in a block of aqueous humor flow into trabecular meshwork. Moreover, lens capsules are broken in mutants (black arrowhead, E) and the lens matrix is found around the lens in the vitreous cavity (black arrow, E). The lens equator in mutants is also totally destroyed and there are very few secondary fiber cells near the lens equator (black broken circle, E). In wild-types, the Schlemm's canal develops well and ciliary body is located between cornea and sclera (arrow, C, WT), whereas in mutants the Schlemm's canal is not obviously found and cornea extends to retina regions (C). (**F**) Retinal rossete-like structure (arrowhead) and retinal traction (arrow) occur in mutants compared with wild types. C, cornea; I, iris; L, lens; R, retina; CE, corneal epithelium; CS, corneal stroma; En, endothelium; CB, ciliary body. Scale bar: A–B, 500 µm; C–D, 50 µm; E (WT), 200 µm; E (a/a, left), 100 µm; E (a/a, right), 50 µm.

To further investigate the eye phenotype of *KTA048* mutants during postnatal periods, we investigated the ocular structure by light microscopy and histology at P21, when major eye morphogenesis is completed. More severe phenotypes are found in *KTA048* mutants compared with the embryonic stages, including smaller eyes and lenses (Fig. 8A), lens-cornea adhesion (Fig. 8A, D and E), thinner corneal epithelium (Fig. 8D) and disorganized corneal stroma (Fig. 8D), missing or very shallow anterior chamber (Fig. 8A and B), iris and ciliary body hypoplasia (Fig. 8C), lens matrix disorganization and disorganized lens epithelium (Fig. 8E) and retinal dysgenesis and retinal retraction (Fig. 8F).

In addition, the lens fibers further leak out not only to the anterior and posterior chambers, but also into the vitreous due to the loss of the structural integrity of the lens epithelium and lens capsule during embryonic development (Fig. 8E). All these phenotypes were found in all mutants at P17–P21 (Table 2). Although the lens stalk was found in 42.9% mutants at E15.5–E17.5 (Table 1), the corneal-lens adhesion was found at P17–P21 in all mutants (Fig. 8D and E; Table 2). These data suggest that the corneal-lens adhesion can be formed at later stages due to a severe dysgenesis of the anterior segment. Such phenotypes of the anterior segment dysgenesis are also a high risk for glaucoma and retinal damage.

**Table 2.** Summary of eye phenotypes in KTA048 homozygous mutants at P17–P21

Phenotype	Disorganized corneal stromal cells	Smaller eyes and lenses	Disorganized lens matrix and lens rupture^a^	Lens tissue in AC and VC	Lens-corneal adhesion	Iris-corneal adhesion	Iris and ciliary body hypoplasia	Vitreous tissue	Retinal folds and rosette	Retinal tract
Number (9 eyes)	9/9	9/9	9/9	9/9	9/9	9/9	9/9	2/9	8/9	8/9
Percentage	100	100	100	100	100	100	100	22.2	88.9	88.9

These data are based on the histological observation of eyes. AC, anterior chamber; VC, vitreous cavity.

^a^Short for the loss of structural identity of the lens capsule.

### Effects on retina and optic nerve head

**Figure 9.**
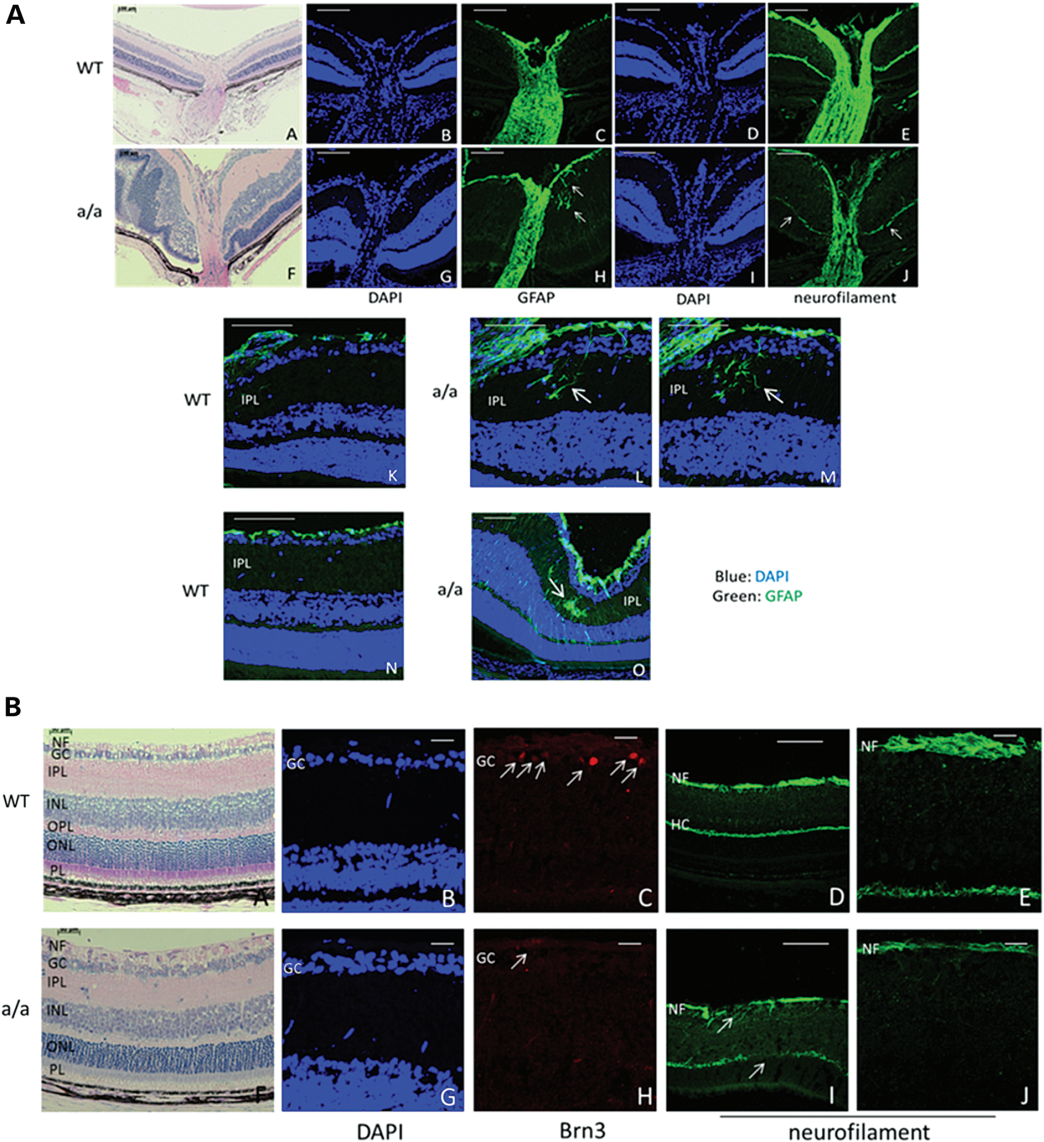
Retina and optic nerve head in *KTA048* mutants. (**A**) (a–j) In the mutants, local retinal folds and rosettes (arrow, f) and retinal detachment (arrowhead, f) are observed. Retinal glial cells are more activated in mutants around the optic nerve head (arrow, h) and in the local inner plexiform layers (h; l–m; o; arrow) and even in full retinal layers of some regions of the mutant retina (o) compared with wild-types (c, h, k and n). There is less staining for neurofilament in the neural fiber layer and optic nerve in mutants (j) compared with wild-types (e). Scale bar: a, f, 100 µm; b–e, g–j, 50 µm. (**B**) (a–c and h–j) There is no dramatic difference in the retinal morphology in well-developed retina of the mutants (f and g) compared with wild-types (a and b), but interestingly the expression of Brn3 is obviously decreased in mutants (h, arrow) compared with wild-types (c, arrow). Correspondingly, the RGC axons in mutants are thinner (i and j) than in wild-types (d and e) and both neural fiber layer and the axons of the horizontal cells are disrupted in mutants (i, arrow; j) compared with wild-types (d and e). GCL, retinal ganglion cell layer; HC, horizontal cells; INL, inner nuclear layer; IPL, inner plexiform layer; NF, nerve fiber layer; ONL, outer nuclear layer; OPL, outer plexiform layer; PL, photoreceptor layer; RGC, retinal ganglion cell. Scale bar: a, b, h, i, d, f, k, m, 50 µm; c, e, g, j, l, n, 20 µm.

Moreover, the retinal phenotypes occur more frequently at postnatal periods (Fig. 8F and Table 2) compared with embryonic stages (Table 1). Not only retinal folds and rosette-like structures (Figs 8F and 9A, F), but also retinal retraction and retinal dysgenesis occur in 88.9% mutants at P17–P21 (Table 2 and Fig. 8F), whereas only local retinal folds and rosette-like structure were found in 32.1% mutants at E15.5–E17.5 (Table 1). These data indicate that retinal dysplasia is progressively severe from embryonic stages to the postnatal periods. Besides, the optic nerve is thinner in mutants compared with wild-types (Fig. 9A, a–j) and neurofilament is less expressed in the optic nerve (Fig. 9A, e and j) and in the retinal nerve fiber (Fig. 9B, d, e, i and j) and is also disrupted in the nerve fiber layer and horizontal cell axons (Fig. 9B, d, e, i and j), suggesting a damage to the optic nerve fibers and axons of the horizontal cells. GFAP is ectopically expressed in the inner plexiform layer in the mutants (Fig. 9A, c, h, l and m), indicating activated glial cells in this region. Some retinal regions showed the activation of retinal glial cells in full retinal layers (Fig. 9A, o). In addition, the number of Brn3-positive retinal ganglion cells is remarkably reduced in the mutants, although in this region the retinal layers are similar to wild-type eyes (Fig. 9B, a–c and f–h), indicating that the retinal ganglion cells are affected in mutants at an early postnatal period (3 weeks). Together, these findings suggest that not only retinal dysplasia, but also the onset and progression of glaucoma occurs in *KTA048* mutants at the early postnatal period.

### Disrupted extracellular matrix and cell adhesion in *KTA048* mutant eyes

**Figure 10.**
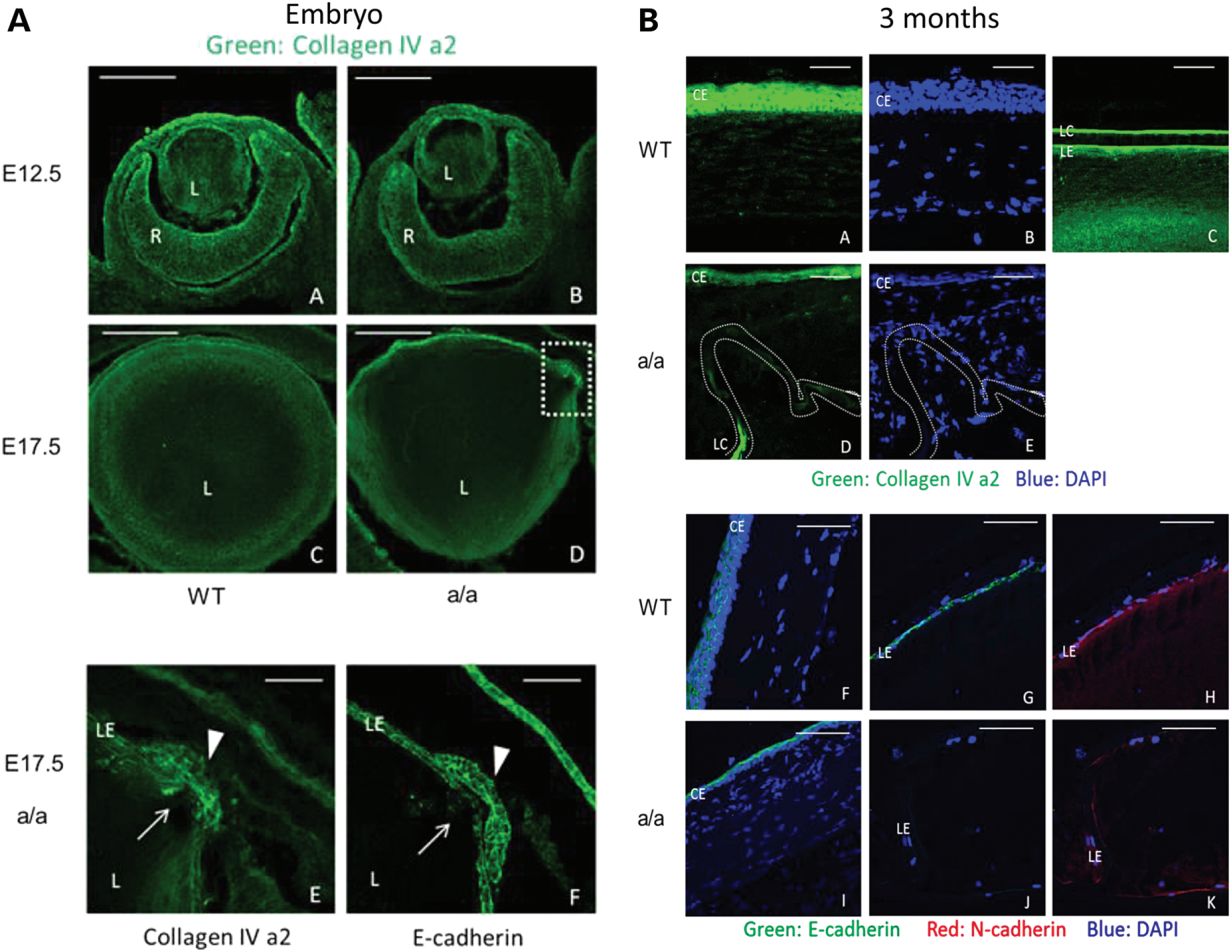
Decreased expression of extracellular matrix molecule and cell adhesion molecule in *KTA048* mutants. (**A**) (a and b) Col4a2 is mainly expressed in the corneal epithelium, lens epithelium and inner limiting membrane in wild-types at E12.5; there is no difference in the expression pattern between wild-types and mutants. (c–e) Local disruption of Col4a2 expression was found in the lens epithelium and lens capsule in mutants (d, broken box: close up in e, f; e, arrow) compared with wild-types at E17.5 (c). E-cadherin immunofluorescence demonstrated less adhesion of lens epithelium cells (f); it appears that lowered cell adhesion of epithelial cells precedes loss of the structural identity of the lens capsule (bulging area, arrow and arrowhead). Scale bar: a–d, 50 µm; e–f, 20 µm. (**B**) (a–e) At 3 months, collagen IV is expressed in the corneal epithelium (a) and lens epithelium and the outer layer of lens capsule (c) in wild-types. The expression of E-cadherin is greatly decreased and disrupted in the cornea epithelium and lens epithelium in mutants (d) in association with disrupted corneal keratocytes (e). (f–k) E-cadherin is expressed in the corneal epithelium (f) and lens epithelium (g), and N-cadherin is expressed in the lens epithelium (h) in wild-types. The expression of E-cadherin and N-cadherin is greatly decreased and disrupted in the cornea epithelium and lens epithelium in mutants (i–k). Scale bar: a–e, 20 µm; f–k, 50 µm.

Like other basement membranes, the lens capsule contains collagen IV, particular the subunits α1, α2, α5 and α6 (30). To investigate the integrity of the lens capsule in the mutants, we used Col4a2 immunofluorescence. In early embryonic eye development, the ocular extracellular matrix was not found to be affected in mutants compared with wild-types at E12.5 (Fig. 10A, a and b). However, besides an obvious loss of structural identity of the lens found in mutants, the local subtle changes including disrupted lens epithelial cell adhesion and lens capsule were also found during embryonic development. Col4a2 immunofluorescence showed locally subtle disrupted lens epithelial cell adhesion and lens capsule in mutants at E17.5 (Fig. 10A, c and d) which also suggests that the lens capsule and lens epithelium adhesion is not consolidated in the mutants; it was confirmed also using E-cadherin immunofluorescence (Fig. 10A, e and f). At adult mice, the extracellular matrix of the anterior segment in the eyes is severely disrupted, which was revealed by Col4a2, E-cadherin and N-cadherin immunofluorescence (Fig. 10B; a–k). In wild-types, Col4a2 is highly expressed in corneal epithelium, the outer layer of lens capsule and lens epithelium (Fig. 10B, a–c), whereas in mutants the expression of Col4a2 is significantly reduced in the corneal epithelium and lens capsule (Fig. 10B, d and e). E-cadherin is also expressed in the corneal epithelium and lens epithelium in wild-types (Fig. 10B, f and g), and N-cadherin is expressed in the lens epithelium (Fig. 10B, h) in adult wild-type eyes. In mutants, the expression of E-cadherin is also obviously decreased in the corneal epithelium cells and lens epithelium cells (Fig. 10B, i and j), and N-cadherin expression is reduced in the lens epithelium cells (Fig. 10B, k). Compared with the embryonic stage, the extracellular matrix and cell adhesion were progressively affected leading to more severe eye damages in adult mutants.

### Genes downstream of *pxdn*

The *KTA048* mutant line is the first mouse model displaying similar eye phenotypes as found in human patients carrying *PXDN* mutations. To identify genes regulated due to reduced expression of *Pxdn* in homozygous mutant mice, whole genome transcriptomics of embryonic eyes at E12.5 were performed. Statistical analysis revealed 486 significantly regulated genes in homozygous embryos compared with wild-type controls (Supplementary Material, Fig. S1). Among the regulated genes, as over-represented functional annotations we found hematological, neurological and developmental diseases as well as post-translational modification, cell death, cell signaling and cellular proliferation (Table 3). Additionally, for several regulated genes expression in the retina, optic disc, optic nerve and cornea or association with cataracts, glaucoma, eye neoplasm and optic nerve disease was described (Supplementary Material, Table S1). Among them, a few genes are up-regulated more than 10-fold [e.g. *Ctsc* (coding for cathepsin C), *Wdr46* (coding for the WD-repeat protein 46) or *Mettl14* (coding for the methyltransferase-like 14 protein)]. On the other side of the extremes, the down-regulation of gene expression is rather moderate. Among these down-regulated genes, *Sncg* (which is represented by two different sequences on the array and is 4-fold down-regulated) might be directly associated with the retinal aspects of the disease. In summary, the *Pxdn* mutation leads to a broad variety of secondary effects as demonstrated by differential expression of developmental genes and genes associated with eye disorders.

**Table 3.** Significantly over-represented functional annotations of regulated genes in eyes of KTA048 mutants

Biological processes	*P*-value	Genes
Hematological disease	7.31E−03	*Add1, Ahsp, Atf4, Ccnd2, Cdk6, Commd3-Bmi1, Ednrb, Fli1, Hbb, Hbd, Hbz, Il6st, Nfe2, Rhag, Slc4a1*
Post-translational modification	6.80E−04	*Bmpr1a, C8orf44-Sgk3/Sgk3, Calr, Ccdc88a, Ccnd2, Ccne2, Cdk6, Cfl1, Dnajc3, Epha3, Ezh2, Fyn, Map2k6, Mapk13, Melk, Pak1, Pkdcc, Prkag2, Prkd3, Ptpn12, Ret, Rock1, Rock2, Sdcbp, Sik1, Sox9, St3gal1, Stk3, Syn1, Thbs1, Wnk1*
Neurological disease	4.98E−04	*Actb, Actn2, Actn3, Arl3, Atp6ap2, B3galt4, B4galt5, Ca11, Capzb, Casp9, Chi3l1, Commd3-Bmi1, Cplx1, Crim1, Eml1, Enpp5, Etv1, Foxn3, Gstp1, Ivns1abp, Lmcd1, Map1b, Mtmr2, Pak1, Pdcl, Ppm1b, Ppp1cb, Prx, Pvalb, Rab5a, Rock2, Rps3a, Rtn1, Sema5a, Sncg, Sox2, Sox9, Sst, St8sia1, Stard10, Stard4, Syn1, Tnnc2, Tnnt1, Ube3a, Wnk1*
Nervous system development	3.44E−04	*Actl6b, Casp9, Ccnd2, Cd200, Celsr3, Chm, Col18a1, Commd3-Bmi1, Crmp1, Dcx, Etv1, Fli1, Folr1, Fyn, Gal, Gli3, Hes5, Hif1a, Il6st, Kif5a, Lef1, Map1b, Mtmr2, Nefm, Nes, Nrn1, Prx, Pvalb, Ret, Rock2, Sall4, Sema5a, Slc17a6, Slc26a5, Slc6a15, Syn1, Ube3a*
Developmental disorder	1.82E−03	*Actc1, Angpt1, Bmpr1a, Csrp3, Ctsc, Dnajc3, Gal, Gucy1a3, Hes5, Hif1a, Il6st, Klk3, Map2k6, Morf4l1, Pnpla8, Rock2, Skp2, Slc4a1, Ttn*
Embryonic development	1.76E−02	*Bmpr1a, Ccne2, Cdk6, Cfl1, Ezh2, Fli1, Hes5, Hif1a, Il6st, Morf4l1, Myl1, Nasp, Nes, Stk3, Ttn*
Cell death and survival	1.62E−02	*Actb, Actc1, Ahsp, Angpt1, Asah1, Atad2, Atf4, Atp6ap2, Atrx, B4galt5, Bcl2l12, Blk, Bmpr1a, Calr, Casp9, Cbfb, Cbx5, Ccdc88a, Ccnd2, Cd200, Cdhr1, Cdk6, Chi3l1, Col18a1, Commd3-Bmi1, Cul4a, Dctn1, Ednrb, Eif3b, Eif4g2, Elavl4, Fah, Fli1, Fyn, Gal, Gcnt1, Gli3, Glo1, Gna13, Gnl3, Gstp1, Hbb, Hif1a, Hspd1, Ifna1/Ifna13, Il6st, Ivns1abp, Klk3, Lef1, Luc7l3, Map2k6, Melk, Mgat3, Msh2, Myo7a, Ncapg2, Pak1, Pawr, Pdcd4, Polh, Ppbp, Ppp1r13b, Prkd3, Pus10, Pvr, Pvrl2, Ret, Rnf41, Rock1, Rock2, Rps3, Rps3a, Rtn1, Scxa/Scxb, Skp2, Slc26a5, Slc4a1, Sncg, Sox9, Srsf5, Sst, St3gal1, Stam, Stk3, Sumo1, Thbs1, Tnc, Tnfrsf11a, Tnfrsf4, Trim10, Ttn, Ube3a*
Cell signaling	1.52E−02	*Atp6ap2, Blk, Fyn, Gna13, Gstp1, Map2k6, Mapk13, Prkag2, Ret, Sik1, Sox2, Stk3, Wnk1, Zdhhc13*
Cellular growth and proliferation	1.42E−03	*Actb, Angpt1, Arl3, Asah1, Asph, Atad2, Atf4, Blk, Bmpr1a, C8orf44-Sgk3/Sgk3, Calr, Casp9, Cbfb, Ccdc88a, Ccnd2, Ccne2, Cdk6, Cfl1, Chi3l1, Col18a1, Commd3-Bmi1, Crk, Ctsc, Cul4a, Cxcr7, Dach1, Dcx, Ddx5, Ednrb, Eif3b, Esm1, Etv1, Ezh2, Fah, Fli1, Folr1, Fyn, Gal, Gcnt1, Gli3, Gnl3, Gstp1, Hes5, Hif1a, Hla-Dqb1, Hnrnpa2b1, Hnrnpf, Hspd1, Ifna1/Ifna13, Il6st, Insig1, Ivns1abp, Klk3, Lef1, Lmo1, Map2k6, Mapk13, Melk, Mgat3, Morf4l1, Nasp, Ncoa4, Nes, Nfe2, Nrn1, Pak1, Pawr, Pdcd4, Polh, Ppp1r13b, Prkd3, Prox1, Pvr, Pxdn, Qpct, Rbm6, Ret, Rnf41, Rock1, Rps3a, Sall4, Scxa/Scxb, Skp2, Slc4a1, Sncg, Sox2, Sox9, Sst, St8sia1, Stam, Stard10, Stk3, Stmn3, Sumo1, Thbs1, Tnc, Tnfrsf11a, Tnfrsf4, Tyrp1, Ube3a, Ugt2b15, Usp9x, Vamp8, Vmp1, Wnk1, Xrn2, Yme1l1, Zc3h12d*

## DISCUSSION

In *KTA048* mutants, an ENU-induced mutant mouse, a nonsense mutation was identified in the *Pxdn* gene; it is predicted that it leads to a premature stop codon affecting the peroxidase domain, causing a loss of function of peroxidase enzyme activity and the loss of the von-Willebrand domain. Deficiency of peroxidasin in this mutant line results in anterior segment dysgenesis and microphthalmia. This is different from most other studies showing that anterior segment dysgenesis is mostly caused by mutations in genes coding for transcription factors, such as *PAX6*, *FOXC1*, *PITX2*, *PITX3*, *Foxe3* and *Ap2α* (2). Compared with the manifestation in patients with *PXDN* mutations presenting congenital cataract, corneal opacity and developmental glaucoma (6), our *peroxidasin*-deficient mice also exhibit highly similar but more severe eye defects, including more severe anterior segment dysgenesis and microphthalmia; frequently, it is associated in postnatal mice with retinal degenerative changes.

The aim of this study was the detailed characterization of the first rodent model suffering from a mutation within the *Pxdn* gene. Besides anterior segment dysgenesis and microphthalmia, the main finding in this study is that peroxidasin has multiple roles during eye development influencing cell proliferation and differentiation and basement membrane consolidation. Ocular inflammation and changes in expression of Pax6 and Foxe3 are understood as secondary effects due to the extrusion of lens material outside the lens, which could be strengthened by the loss of peroxidase activity in the mutant mice, which might cause an early degenerative damage in the retina and the optic nerve.

Obviously, peroxidasin does not affect early eye development, because at E9.5–E12.5, the early stages for eye development in mice, *peroxidasin*-mutants do not exhibit gross eye morphological changes. However, especially at E15.5, around middle stage for eye development in mice, *KTA048* mutants showed obvious eye phenotypes, including anterior segment dysgenesis and smaller lens, particularly the loss of the structural identity of the lens capsule and the lens epithelium adhesion which results in lens fibers leaking into the anterior and posterior chambers and lens disorganization in mutants. In some cases at E15.5–E17.5 (43%), persistent corneal-lens adhesion was found, which is associated with corneal disorganization. However, corneal-lens adhesion was found in nearly all the mutant mice at the postnatal periods and all mutant showed corneal opacity but vary in clinical severity. These observations provide a general pathological developmental process leading to a delayed and abnormal eye development. We found that *Pax6*, the master gene for eye development (25,26), underwent dynamic expression changes in *peroxidasin*-mutant eyes, which is strongly up-regulated at E15.5, the stage with the most obvious pathological changes, and recover to the normal level at P10. Overexpression of *Pax6* in the mouse eye can also affect the normal development of anterior segment, resulting in similar eye phenotypes to *KTA048* mutants, including corneal opacity (31–33), iris hypoplasia (31), abnormal lens fiber cell differentiation (32), microphthalmia (31,33) and retinal dysplasia (34). In addition, lens growth retardation is prior to ocular growth retardation, since the lens size is smaller in mutants than controls but no statistical difference in ocular size was found at E15.5. Aphakia (absence of the lens) can cause microphthalmia in mice (35). Thus, the microphthalmia exhibited in *KTA048* mutants is likely the result of the retarded lens growth.

Due to reduced lens size in *peroxidasin*-mutant eyes during embryonic development, the mutant eyes could undergo decreased proliferation and disrupted differentiation. To test this hypothesis, *in vivo* BrdU labeling demonstrated a decreased number of proliferating cells in the lens epithelium compared with wild-types at E14.5 and E15.5, suggesting a decreased cell proliferation during development. Meanwhile, caspase-3 positive cells were not detected in the mutant embryonic eyes to exclude the apoptosis effect (data not shown). Thus, the anterior epithelium cells failed to produce more fiber cells so that the lens cannot grow to the normal size. Correspondingly, Foxe3, a molecule which is necessary for lens cell proliferation and differentiation (36,37), is expressed at a lower level in the lens epithelium in the mutant eyes at E15.5, E17.5 and P11; it is also ectopically expressed in the posterior chamber and vitreous at P11 due to the lens fiber cells enter into these places through the loss of the structural identity of the lens capsule and lens epithelium adhesion. Together, these results demonstrate that peroxidasin is required for regulation of cell proliferation in the lens during development. On the other hand, lens differentiation is also affected in *peroxidasin*-mutant eyes. The primary fiber cells, which are differentiated from posterior lens epithelial cells and elongate to fill the lens vesicle, are not affected during early eye development before E14.5. But subsequently, the secondary fiber cells which are differentiated from the lens epithelial cells at the transitional zone of the lens are significantly reduced at E15.5. Postnatally, at P21, less secondary fiber cells with their dramatic changes in cell shape are present in the mutants. Together, peroxidasin is also essential for lens fiber cells differentiation.

Consistent with an important role of peroxidasin in the formation and consolidation of the extracellular matrix in *Drosophila* and *C. elegans* (12,14), we also observed that there are dramatic changes in the extracellular matrix in all *peroxidasin*-mutant eyes during embryonic development. Particularly, the loss of the structural identity of the lens capsule and lens epithelium adhesion occurs in embryonic eyes, and they are more severe in adult. A recent study demonstrated that peroxidasin crosslinks collagen IV by forming sulfilimine bonds to maintain tissue integrity (15). Collagen IV, the main component of basement membrane, is mainly expressed in the lens capsule (30) and highly expressed in the inner limiting membranes (38,39) during eye development. In our results, collagen IV expression is decreased in the lens epithelium of embryonic eyes and in the adult lens. In addition, a loss of structural integrity of the inner limiting membrane is also observed in some mutants. These changes could be due to the failure to crosslink collagen IV by the mutant peroxidasin which thereby destabilizes the ocular basement membrane. Thus, these data confirmed that peroxidasin is involved in ECM consolidation in a higher organism and also showed that peroxidasin is essential for the functional integrity of basement membranes and cell adhesion during embryonic eye development.

The decreased expression of collagen IV in our *Pxdn*-mutants is the reason why they display abnormalities strikingly reminiscent of some *Col4a1* mouse mutants affecting also ocular basal membranes. Pathologies in *Col4a1* mutants include iris defects, corneal opacities, vacuolar cataracts and significant iris/corneal adhesions (40), disorganization of the lens epithelial layer together with vacuoles in the underlying secondary fiber cell region and corneal-lens adhesion (41) or corneal opacity, enlarged and torturous iris vasculature, cataracts and iridocorneal adhesion (42,43). It has to be mentioned that the severity of the ocular defects varies among the different *Col4a1* alleles, but there is also some phenotypic variability within each mutant line—similar to the features observed in our *Pxdn* mutants as outlined in Tables 1 and 2. In addition, mutations in *Col4a1* (44–46) and *Col4a2* (47) can cause hemorrhagic stroke, but similar changes in *Pxdn* mutants were not found.

Similarly, the white spot at the belly of our *Pxdn* mutant is reminiscent of some mutations affecting migration of neural crest cells (reviewed in 48). In these cases, the white spot indicates a failure in neural crest cell migration; neural crest cell migration is also involved in several aspects during eye development (reviewed in 49) and might contribute to iris hypoplasia.

One of the major consequences in our *Pxdn* mutants is the ocular inflammation. Since lens fiber cells need to grow and elongate, they were extruded in the mutants out of the lens into the anterior and posterior chambers and vitreous cavity through the loss of structural identity of the lens capsule and lens epithelium adhesion. The lens cells in the anterior chamber also worsen the development of the anterior segment and can delay eye development in general. Moreover, the crystallins in the anterior chamber might induce ocular inflammation and glaucoma (50,51). During this embryonic stage (E15.5) and the early postnatal period (P10) before eye opening, genes representing inflammation markers such as *IL-1β* and *TNF-α* are significantly up-regulated suggesting that congenital ocular inflammation occurs. Meanwhile, the ocular inflammation could also be strengthened by a reduced peroxidase activity caused by a nonsense mutation in the peroxidase domain of peroxidasin.

Many risk factors for glaucoma were found in *KTA048* mutants during eye development, including severe anterior segment dysgenesis, smaller eyes, and iris-corneal adhesion, lens-corneal adhesion and lens crystallin in the anterior chamber. Therefore, *KTA048* mutants could have glaucoma, although it is very difficult to measure ocular pressure in these mutant mice due to nearly closed eye lids. The hallmark of glaucoma is the damage of optic nerve head and retina, including loss of retinal ganglion cells and their axons (52). We found that the expression of Brn3 (retinal ganglion cell marker) at 3 weeks is significantly decreased in the retina, suggesting that RGCs are damaged. Moreover, the nerve fibers, consisting of retinal ganglion cell axons, are disrupted in the retina and also are much thinner in the retina and optic nerve head in mutants than in normal controls, suggesting that the retinal ganglion cells axons are degenerative in mutants as early as 3 weeks. Together, these findings indicate that early glaucoma occurs in the *KTA048* mice and it is congenital glaucoma due to anterior segment dysgenesis during embryonic periods. Therefore, peroxidasin could play a role in the onset of eye inflammation and congenital glaucoma.

In summary, our study suggests that peroxidasin is essential for eye development including proliferation and differentiation, which could be mediated by regulation of *Pax6*. In addition, peroxidasin is also essential for basement membrane integrity and cell adhesion during eye development. The mutant eyes exhibit congenital eye inflammation and congenital glaucoma hallmarks. Our studies also provide insights for the eye defects found in the patients with *PXDN* mutations, which could aid in the development of novel strategies to treat congenital anterior segment dysgenesis including corneal opacity, cataract and glaucoma.

## MATERIALS AND METHODS

### Mice

Mice were kept under specific pathogen-free conditions at the Helmholtz Center Munich. The use of animals was in accordance with the German Law of Animal Protection, the ARVO Statement for the Use of Animals in Ophthalmic and Vision Research, and the tenets of the Declaration of Helsinki; it was approved by the Government of Upper Bavaria under the registration number 55.2-1-54-2532-126-11. Male C3HeB/FeJ (C3H) mice were treated with ENU (90 mg/kg body weight applied by intraperitoneal injection in three weekly intervals) at the age of 10–12 weeks as previously described (19,53) and mated to untreated female C3H mice. The offspring of the ENU-treated mice were screened at the age of 11 weeks for dysmorphological parameters.

Since the recessive eye phenotype was observed in hybrids of C3H and C57BL/6J (B6) mice, we established two sub-lines by brother × sister mating, one using brown mice reflecting the C3H background, and the other using black mice reflecting the B6 background. The sub-lines have been kept for 4 years as homozygous brother × sister mating before the experiments reported here have been started. For Figures 2–7 and for Figure 10, we used the sub-line on C3H background and correspondingly C3H mice from our own breeding colony as wild-type controls. For Figures 8 and 9, we used the B6 mice and correspondingly B6 mice from our own colony as wild-type controls.

### Linkage analysis

Heterozygous carriers (first generation) were mated to wild-type (B6) mice, and the offspring (second generation) were again backcrossed to wild-type B6 mice. DNA was prepared from tail tips of affected offspring of the third generation (G3). For linkage analysis, genotyping of a genome-wide mapping panel consisting of 153 SNPs was performed using MassExtend, a MALDI-TOF (matrix-assisted laser/desorption ionization, time of flight analyzer) mass spectrometry high-throughput genotyping system supplied by Sequenom [San Diego, CA, USA (54)].

### Genotyping and sequencing

RNA was isolated from embryonic mouse eyes (E15.5) and reverse transcribed to cDNA using the Ready-to-Go T-primed first strand kit (Invitrogen, Germany). Genomic DNA was isolated from tail tips of C3H, B6, CFW, DBA/2J and JF1 wild-type mice or homozygous/heterozygous embryos (E15.5; on C3H background) according to the standard procedures. PCR was performed with a Flex Cycler (Analytik Jena, Jena, Germany) using primers and conditions as listed in Table 4. Products were analyzed by electrophoresis on a 1.5% agarose gel. Sequencing was performed commercially (GATC Biotech, Konstanz, Germany) after direct purification of the PCR products (Nucleospin Extract II, Macherey-Nagel, Düren, Germany). To confirm the mutation in the genomic DNA, a 356-bp fragment was amplified from genomic DNA using the primer pair *Pxdn-Ex19-L1* and *Pxdn-Ex19-R1* (Table 4); in the presence of the mutation, this fragment can be digested by the restriction endonuclease *Alw*26I into two fragments of 145 and 211 bp.

**Table 4.** List of PCR primers

Lab-No	Sequence (5′–> 3′)	Fragment size	Annealing temperature
*Pxdn-L1*	AGGGCTCAGTTGGGAGCC	900	65°C
*Pxdn-R1*	TTGGGGTTGCCCTCAGC		
*Pxdn-L2*	CATGCGAGTATCCAGACGC	1020	66°C
*Pxdn-R2*	CAACATCATTGATGGTCAAGAATCC		
*Pxdn-L3*	AGTCACCCCGGTATTTGCC	1140	60°C
*Pxdn-R3*	ATGAGCAGGGACGTCATGC		
*Pxdn-L4*	AGCAGTTTACACACATGCTGATGC	1120	66°C
*Pxdn-R4*	AGGTCCTCAAAGGTGTAAGCAGC		
*Pxdn-L5*	ACCCACTTCTCCGAGGGC	780	66°C
*Pxdn-R5*	CATTAGTTGCTGGCCCTTCC		
*Pxdn-L6*	TTATAGCAGCTGTGAGGACATCCC	760	66°C
*Pxdn-R6*	AGTGAGGGCCAGAGCCTGC		
*Pxdn-Ex19-L1*	CCTTGTGGCTGACATTCTCCC	356	55°C
Pxdn-Ex19-R1	CACTTTCCCCGTTCTCAGGC		

### Real-time PCR

RNA was extracted using RNeasy mini kit (Qiagen, Germany) according to the manufacture's instruction. cDNA was synthesized using Ready-To-Go T-primed first strand kit (Invitrogen, Germany). qRT-PCR was performed with StepOne^TM^ Real-Time PCR System (Applied Biosystems, Germany). The primers were standardized and the efficiency was tested before performing real-time PCR; primers with an efficiency above 90% were used in this study. In each reaction, 1 μl cDNA, 1 μl reverse and forward primers (1:1), 4 μl EvaGreen mix and 14 μl DEPC-H_2_O were mixed in one well in a 96-well plate and centrifuged briefly. After the initial denaturation step at 95°C for 15 min, PCR reaction was cycled for 40 times with denature at 95°C for 30 s and annealing-extension temperature at 65°C for 30 s. Relative expression was calculated following the 2(-Delta Delta C(T)) method (55). Primers are listed in Table 5.

**Table 5.** Primers for real-time RT-PCR

Gene	Primer	Sequence (5′–> 3′)
*Pax6*	Pax6-qF	GTTCTTCGCAACCTGGCTA
	Pax6-qR	TGAGCTTCATCCGAGTCTTCT
*TNF-α*	TNFα_2F	CACCACGCTCTTCTGTCT
	TNFα_2R	GGCTACAGGCTTGTCACTC
*IL-1β*	IL-1β_FW	CAACCAACAAGTATTCTCCATG
	IL-1β_RV	GATCCACACTCTCCAGCTGCA
*Tubea*	TubeaF	CCAGATGCCAAGTGACAAGA
	TubeaR	GTGGGTTCCAGGTCTACGAA

### Eye morphology and function

To get the embryos, respective animals were bred and the vaginal plug was used to detect the pregnancy. The noon of positive plug day was used as *post coitum* day 0.5 and the females were sacrificed in a CO_2_ chamber around noon of the respective post coitum days to collect the embryos.

For histological analysis, the heads of the embryos were fixed in Davidson's solution overnight, dehydrated in 100% ethanol for three times (each for 15 min) and embedded in JB-4 plastic medium (Polysciences Inc., Eppelheim, Germany) according to the manufacturer's protocol. Sectioning was performed with an ultramicrotome (OMU3; Reichert-Jung, Walldorf, Germany). Serial transverse 3 µm sections were cut with a glass knife and stained with methylene blue and basic fuchsin as described previously (56).

For anterograde tracing, E15.5 embryos were fixed in 4% PFA in PBS overnight, and the cornea and lens were removed and the optic cup was packed with DiI, a fluorescent lipophilic cationic indocarbocyanine dye. The embryos were refixed in 4% PFA in PBS for 3 weeks at 37°C to allow the tracers to diffuse along axons completely. The optic nerve, optic chiasm and optic tract were exposed after removing the heads, and analyzed under the stereo-fluorescence microscopy.

For morphometric analysis, the middle sections of the eyes stained by methylene blue and Basic fuchsin were used to measure eye size, lens size and the depth of anterior chamber at E15.5, E17.5 and P21. The length between the central corneal epithelium and the central point of the optic nerve head was measured as eye size, and the length between the anterior central point of anterior lens capsule and posterior lens capsule was measured as lens size. The depth of anterior chamber was measured between the central point of corneal endothelium and the central point of the anterior lens capsule.

For immunofluorescence, embryos were fixed in 4% PFA overnight and processed for cryosection or paraffin embedding and sectioned. For cryosectioning, embryos were cryoprotected in 30% sucrose solution in PBS, embedded in OCT (Sakura, Torrance, USA) and sectioned at 12 μm. For paraffin embedding, embryos were first dehydrated in serial dilution of methanol, followed by bleaching in 3% H_2_O_2_ for 1 h, washed twice in absolute methanol for 10 min each, embedded in paraffin and sectioned at 8 μm by RM 2065-*microtome* (Leica, Wetzlar, Germany).

Embryonic cryosections or paraffin sections were washed in PBS and the paraffin sections were deparaffinized in Roti-Histol (Roth, Karlsruhe, Germany) followed by rehydration in descending ethanol series. For antigen retrieval in paraffin sections, sections were boiled in 0.01 m sodium citrate buffer (pH 6.4) and cooled slowly by adding MilliQ water. Cryo-sections were processed without an antigen retrieval step. Tissue sections were treated with 3% fetal bovine serum in PBS containing 0.25% Triton X-100 (for blocking) and incubated with the primary antibody(ies) at 4°C for overnight. After washing in PBS, sections were incubated with the appropriate secondary antibody for 90 min, counterstained with DAPI and mounted using Aqua-Poly/Mount (Polysciences). Images (single plane images and Z-stacks) were obtained with an Olympus confocal microscope (Hamburg, Germany) and analyzed by a FluoView software (Olympus). Antibodies are given in Table 6.

**Table 6.** Primary antibodies

Target protein	Species	Dilution	Catalog no.	Company
BrdU	Rat	1:500	OBT0030CX	AbD Serotec, Germany
Brn3	Goat	1:100	Sc-6026	Santa Cruz, Germany
Col4A2	Rabbit	1:200	sc-70246	Santa Cruz, Germany
E-cadherin	Rat	1:200	U3254	Sigma-Aldrich, Germany
Foxe3	Rabbit	1:200	Sc-134536	Santa Cruz, Germany
GFAP	Rabbit	1:500	G9269	Sigma-Aldrich, Germany
N-cadherin	Rabbit	1:250	Sc-7939	Santa Cruz, Germany
Neurofilament 200	Rabbit	1:500	N4142	Santa Cruz, Germany
Pax2	Rabbit	1:200	2549-1	Epitomics, Germany
Pax6	Rabbit	1:400	PRB-278P	Chemicon, Germany
PXDN	Rabbit	1:500	A gift from Dr Gautam Bhave, Vanderbilt University Medical Center, USA	
γ-Crystallin	Rabbit	1:200	Sc-22746	Santa Cruz, Germany
Secondary antibodies				
Alexa Fluor^®^ 488	Rabbit	1:250	A21206	Invitrogen, Germany
Alexa Fluor^®^ 488	Rat	1:250	A21208	Invitrogen, Germany
Cy3	Goat	1:250	705-165-147	Dianova, Germany
Cy5	Mouse	1:250	715-175-150	Jackson Immuno, Germany

### BrdU labeling

To label the dividing cells with the thymidine analog, 5-bromo-2′-deoxy-uridine (BrdU), pregnant mice were injected peritoneally with BrdU solution at a concentration of 0.1 mg/g body weight on the required embryonic day. Two hours following BrdU injection, mice were sacrificed and embryos were collected, fixed in 4% PFA, cryoprotected in 30% sucrose and embedded in Tissue-Tec OCT (Hartenstein, Würzburg, Germany). Tail tips were collected to genotype the embryos. BrdU was detected by immunofluorescence staining.

### Transcriptome analysis of eyes

Eyes of E12.5 embryos (homozygote *n* = 8, wild-type *n* = 8) were dissected and four eyes of two embryos of the same genotype combined to one sample. Total RNA of pooled samples was extracted according to a standardized protocol (RNAeasy mini kit, Qiagen). Illumina MouseRef 8v2 Expression Bead Chips were conducted as previously described (57) and data normalization performed with Illumina Genomestudio 2011.1 (cubic spline normalization, background subtraction). Statistical analysis to identify differential gene expression in mutant tissue compared with wild-type was performed using SAM [Significant Analysis of Microarrays (58–60)] (FDR < 10%, mean fold change > 1.5). Overrepresented functional annotations within the data set were provide as GO (Gene Ontology) terms of the category biological processes (ingenuity pathway analysis, IPA). The complete array data set is available from the GEO database (61) under accession number GSE49704.

### Statistics

The two sample *t*-test was used to compare the means of two groups. If the variance was not equal and confirmed by *F*-test, a non-parametric Mann–Whitney test was used for further statistical analysis. If *P* *<* 0.05, it is reported as statistically different.

### General

Chemicals and enzymes were from Fermentas (St-Leon-Rot, Germany), Merck (Darmstadt, Germany) or Sigma Chemicals (Deisenhofen, Germany). Oligonucleotides were synthesized by Sigma Genosys (Steinheim, Germany).

## SUPPLEMENTARY MATERIAL

Supplementary Material is available at *HMG* online.

## FUNDING

This project was supported by a fellowship from China Scholarship Council to X.Y. This project was also supported at least in part by grants from the Federal Ministry of Education and Research within the framework of the NGFN (NGFN-Plus; grant identifications: BMBF 01KW9923, and BMBF 01GS0850) and Infrafrontier (01KX1012). Additional funding was provided by the European Union (EUMODIC LSHG-2006-037188 and Infrafrontier Contract No. 211404). Funding to pay the Open Access publication charges for this article was provided by Helmholtz Center Munich.

## Supplementary Material

Supplementary Data
