# Supplementary material for: Peroxidasin is essential for eye development in the mouse
Source: Hum Mol Genet. 2014 Jun 3;23(21):5597–614. doi: 10.1093/hmg/ddu274 (PMC4189897; doi:10.1093/hmg/ddu274)
Supplement: Supplementary Data [file supp_23_21_5597__index.html]

Peroxidasin is essential for eye development in the mouse — Peroxidasin is essential for eye development in the mouse — Supplementary Data 

# Peroxidasin is essential for eye development in the mouse

## Supplementary Data

Supplementary Data

**Files in this Data Supplement:**

- Supplementary Data - Pdf file
