# Supplementary material for: Peroxidasin is essential for eye development in the mouse
Source: Hum Mol Genet. 2014 Jun 3;23(21):5597–614. doi: 10.1093/hmg/ddu274 (PMC4189897; doi:10.1093/hmg/ddu274)
Supplement: Supplementary Data [file supp_ddu274_ddu274supp.pdf]

Supplementary Figure S1

Mean linear fold change	*Pxdn* mutant mice A	B	C	D	Illumina ID	Gene symbol	Gene name
1.75					ILMN_1220586	Papd4	PAP associated domain containing 4
1.86					ILMN_1246639	Slc26a5	solute carrier family 26, member 5
1.76					ILMN_3055904	Cbx5	chromobox homolog 5
1.86					ILMN_2770183	Ddah2	dimethylarginine dimethylaminohydrolase 2
1.86					ILMN_1226520	Angpt1	angiopoietin 1
1.80					ILMN_2720836	Rbm47	RNA binding motif protein 47
1.81					ILMN_2615369	Dnajc18	DnaJ (Hsp40) homolog, subfamily C, member 18
1.77					ILMN_1237736	Plekhf2	pleckstrin homology domain containing, family F, member 2
1.77					ILMN_1258813	Golim4	golgi integral membrane protein 4
1.72					ILMN_1247242	Sdccag1	serologically defined colon cancer antigen 1
1.85					ILMN_2730424	Ugt2b38	UDP glucuronosyltransferase 2 family, polypeptide B38
1.85					ILMN_2691815	G3bp2	GTPase activating protein (SH3 domain) binding protein 2
1.70					ILMN_3013874	Gm5643	
1.78					ILMN_2638778	Luc7l3	LUC7-like 3
1.91					ILMN_2706809	Spata3	spermatogenesis associated 3
1.97					ILMN_2663139	Pknox2	Pbx/knotted 1 homeobox 2
1.92					ILMN_2604298	A730049H05Rik	
1.73					ILMN_3063644	Syt11	synaptotagmin XI
2.04					ILMN_2648475	Lef1	lymphoid enhancer binding factor 1
1.79					ILMN_2645493	Pvrl2	poliovirus receptor-related 2
1.78					ILMN_2706176	Glcci1	glucocorticoid induced transcript 1
1.80					ILMN_2731760	Myo1f	myosin IF
1.70					ILMN_2829171	Hnrnpf	heterogeneous nuclear ribonucleoprotein F
1.86					ILMN_2655183	Lcor	ligand dependent nuclear receptor corepressor
1.79					ILMN_2688923	Hnrnph1	heterogeneous nuclear ribonucleoprotein H1
1.98					ILMN_2863060	Ccdc109a	coiled-coil domain containing 109A
1.86					ILMN_3084087	Ivns1abp	influenza virus NS1A binding protein
1.81					ILMN_2680128	Zc3h13	zinc finger CCCH type containing 13
1.73					ILMN_2750548	Gnpda1	glucosamine-6-phosphate deaminase 1
1.77					ILMN_2997256	Pdcl	phosducin-like
1.89					ILMN_2642705	Ier2	immediate early response 2
1.98					ILMN_3151210	Chm	choroidermia
2.15					ILMN_2720338	Dach1	dachshund 1
1.73					ILMN_1256671	Hnrnph1	heterogeneous nuclear ribonucleoprotein H1
1.89					ILMN_2935909	Bcl2l12	BCL2-like 12
1.75					ILMN_2689940	Dhdh	dihydrodiol dehydrogenase
1.73					ILMN_1214110	Rnf8	ring finger protein 8
2.33					ILMN_2482600	Tnfrsf11a	tumor necrosis factor receptor superfamily, member 11a
2.10					ILMN_2663821	Fyn	Fyn proto-oncogene
1.99					ILMN_2760057	BC068281	
1.82					ILMN_1240445	Stard4	StAR-related lipid transfer (START) domain containing 4
1.75					ILMN_2678495	Akap9	A kinase (PRKA) anchor protein 9
1.78					ILMN_2681057	Phca	alkaline ceramidase 3
1.80					ILMN_2750740	Flrt3	fibronectin leucine rich transmembrane protein 3
1.85					ILMN_1246060	Klhl9	kelch-like 9
1.99					ILMN_1215183	Gli3	GLI-Kruppel family member GLI3
1.85					ILMN_1241795	Gpbp1l1	GC-rich promoter binding protein 1-like 1
1.93					ILMN_2628657	Atp6ap2	ATPase, H+ transporting, lysosomal accessory protein 2
2.01					ILMN_1255301	2410042D21Rik	
1.94					ILMN_2703032	Eny2	enhancer of yellow 2 homolog (Drosophila)
1.86					ILMN_2630039	E130014J05Rik	
1.85					ILMN_1215722	Zdhhc13	zinc finger, DHHC domain containing 13
2.17					ILMN_2751653	Pnpla8	patatin-like phospholipase domain containing 8
1.74					ILMN_1214800	Rnf167	ring finger protein 167
1.75					ILMN_2680160	Nemf	nuclear export mediator factor
1.77					ILMN_3111334	Rsrc2	arginine/serine-rich coiled-coil 2
1.86					ILMN_2717765	Ednrb	endothelin receptor type B
1.79					ILMN_2657911	Cnot4	CCR4-NOT transcription complex, subunit 4
2.50					ILMN_2671698	Epas1	endothelial PAS domain protein 1
2.38					ILMN_2748300	2010012O05Rik	

1.91					ILMN_3160771	Cd200	CD200 antigen
1.78					ILMN_2756113	Nln	neurolysin (metallopeptidase M3 family)
1.81					ILMN_2759762	Stard10	START domain containing 10
1.80					ILMN_2708397	Rsl24d1	ribosomal L24 domain containing 1
1.83					ILMN_2629804	Epha3	Eph receptor A3
1.78					ILMN_2786764	Rab5a	RAB5A, member RAS oncogene family
2.13					ILMN_2693858	D14Ertd449e	DNA segment, Chr 14, ERATO Doi 449, expressed
1.84					ILMN_2649709	Etv1	ets variant gene 1
1.89					ILMN_1226901	Man2a2	mannosidase 2, alpha 2
1.84					ILMN_1220596	Ifna5	interferon alpha 5
2.29					ILMN_2512442	Zfp395	zinc finger protein 395
2.02					ILMN_2441921	Trim56	tripartite motif-containing 56
1.84					ILMN_2643915	Etv1	ets variant gene 1
1.83					ILMN_1240529	Cdh11	cadherin 11
1.89					ILMN_1241458	Arap1	ArfGAP with RhoGAP domain, ankyrin repeat & PH domain 1
1.86					ILMN_2668805	Stam	signal transducing adaptor molecule 1
1.98					ILMN_2425003	Tyrp1	tyrosinase-related protein 1
1.86					ILMN_2756286	Larp5	La ribonucleoprotein domain family, member 5
1.95					ILMN_1240267	Hba-x	hemoglobin X, alpha-like embryonic chain in Hba complex
1.94					ILMN_1229680	Flrt3	fibronectin leucine rich transmembrane protein 3
1.90					ILMN_1255836	Tgs1	trimethylguanosine synthase homolog
2.05					ILMN_2694758	Efr3a	EFR3 homolog A
1.79					ILMN_2661107	Atl2	atlastin GTPase 2
1.98					ILMN_1245789	Ccne2	cyclin E2
2.06					ILMN_2650511	Lcor	ligand dependent nuclear receptor corepressor
1.78					ILMN_2590321	Nhlrc2	NHL repeat containing 2
2.02					ILMN_2680808	Hnrnph2	heterogeneous nuclear ribonucleoprotein H2
1.90					ILMN_3155041	Lrba	LPS-responsive beige-like anchor
2.24					ILMN_2752146	Abat	4-aminobutyrate aminotransferase
2.14					ILMN_2686594	Lix1	limb expression 1 homolog
1.82					ILMN_2656102	Sox2	SRY-box containing gene 2
1.84					ILMN_2719888	G3bp1	Ras-GTPase-activating protein SH3-domain binding protein 1
1.81					ILMN_2663555	Asb3	ankyrin repeat and SOCS box-containing 3
1.87					ILMN_2740464	Il1rap	interleukin 1 receptor accessory protein
2.15					ILMN_1257143	Ermap	erythroblast membrane-associated protein
1.89					ILMN_1248340	Lsg1	large subunit GTPase 1 homolog
2.00					ILMN_2641660	B4galt5	betaGlcNAc beta 1,4-galactosyltransferase, polypeptide 5
1.99					ILMN_2704467	Smarcd1	SWI/SNF related, actin dependent regulator of chromatin, d1
1.99					ILMN_2719131	U2surp	U2 snRNP-associated SURP domain containing
1.90					ILMN_2503651	Usp9x	ubiquitin specific peptidase 9, X chromosome
1.82					ILMN_2599748	Map2k6	mitogen-activated protein kinase kinase 6
1.86					ILMN_2729953	Ppm1b	protein phosphatase 1B, magnesium dependent, beta isoform
1.79					ILMN_3045217	Setd2	SET domain containing 2
1.94					ILMN_2826414	Wdfy3	WD repeat and FYVE domain containing 3
2.03					ILMN_2499400	Vamp8	vesicle-associated membrane protein 8
1.95					ILMN_3038459	Morf4l1	mortality factor 4 like 1
2.09					ILMN_1254531	Phf20l1	PHD finger protein 20-like 1
1.82					ILMN_2616768	Tmem49	transmembrane protein 49
2.07					ILMN_2683958	Col3a1	collagen, type III, alpha 1
2.00					ILMN_1231967	Zcchc11	zinc finger, CCHC domain containing 11
3.40					ILMN_2994806	H2afj	H2A histone family, member J
1.91					ILMN_1233746	Eif3b	eukaryotic translation initiation factor 3, subunit B
1.84					ILMN_2669172	4930547N16Rik	
1.93					ILMN_1225204	Rsad2	radical S-adenosyl methionine domain containing 2
1.98					ILMN_2665359	Sgk3	serum/glucocorticoid regulated kinase 3
2.31					ILMN_1251682	Prei4	preimplantation protein 4
1.93					ILMN_2673441	Gnl3	guanine nucleotide binding protein-like 3
1.95					ILMN_2746116	Osta	organic solute transporter alpha
1.86					ILMN_2705848	Decr2	2-4-dienoyl-Coenzyme A reductase 2, peroxisomal
1.92					ILMN_2482766	Tspyl1	testis-specific protein, Y-encoded-like 1
1.87					ILMN_3157232	Zc4h2	zinc finger, C4H2 domain containing
2.12					ILMN_1228102	Ppbp	pro-platelet basic protein
1.99					ILMN_2628658	Atp6ap2	ATPase, H+ transporting, lysosomal accessory protein 2
1.94					ILMN_2750248	Mtap1b	microtubule-associated protein 1B
1.98					ILMN_3137570	Pdlim5	PDZ and LIM domain 5
1.91					ILMN_3149251	Lgsn	lengsin, lens protein with glutamine synthetase domain

1.92					ILMN_1229706	Ammecr1l	AMME chromosomal region gene 1-like
1.97					ILMN_3046836	Suclg2	succinate-Coenzyme A ligase, GDP-forming, beta subunit
2.04					ILMN_2771219	Pdlim5	PDZ and LIM domain 5
2.11					ILMN_3145881	Cnot2	CCR4-NOT transcription complex, subunit 2
1.95					ILMN_1249093	St8sia1	ST8 alpha-N-acetyl-neuraminide alpha-2,8-sialyltransferase 1
1.93					ILMN_1236125	Mcm6	minichromosome maintenance deficient 6
1.90					ILMN_2680648	Atad2	ATPase family, AAA domain containing 2
2.23					ILMN_3154869	Ctdspl2	CTD small phosphatase like 2
2.11					ILMN_2605756	Klc1	kinesin light chain 1
1.98					ILMN_1254179	Rap2b	RAP2B, member of RAS oncogene family
2.08					ILMN_2619249	Snx14	sorting nexin 14
1.96					ILMN_1258914	Slc6a15	solute carrier family 6, member 15
1.99					ILMN_1240566	Cep170	centrosomal protein 170
2.29					ILMN_2621122	Ube2q2	ubiquitin-conjugating enzyme E2Q (putative) 2
2.03					ILMN_3150724	Ppfibp1	PTPRF interacting protein, binding protein 1
1.98					ILMN_1221405	Crk	v-crk sarcoma virus CT10 oncogene homolog
2.14					ILMN_2423988	Zfp113	zinc finger protein 113
2.03					ILMN_2684986	Hnrph1	heterogeneous nuclear ribonucleoprotein H1
1.93					ILMN_3075157	Tdrd3	tudor domain containing 3
1.96					ILMN_1248041	Rock2	Rho-associated coiled-coil containing protein kinase 2
1.96					ILMN_2780022	4833424O15Rik	
1.89					ILMN_1231392	Melk	maternal embryonic leucine zipper kinase
1.92					ILMN_2687561	Cxcr7	chemokine (C-X-C motif) receptor 7
1.96					ILMN_1215521	Map2k6	mitogen-activated protein kinase kinase 6
2.02					ILMN_2669343	Sass6	spindle assembly 6 homolog
1.96					ILMN_2669461	Bbx	bobby sox homolog
2.04					ILMN_1229173	H2-T24	histocompatibility 2, T region locus 24
1.98					ILMN_2632971	B3galt4	UDP-Gal:betaGlcNAc beta 1,3-galactosyltransferase, 4
2.03					ILMN_1247897	Lix1	limb expression 1 homolog
2.17					ILMN_2506499	Ube3a	ubiquitin protein ligase E3A
2.05					ILMN_1252098	Pik3c2a	phosphatidylinositol 3-kinase, C2 domain containing, alpha
1.99					ILMN_2589477	Dars	aspartyl-tRNA synthetase
2.14					ILMN_1225839	Col18a1	collagen, type XVIII, alpha 1
2.04					ILMN_2757855	Ehmt1	euchromatic histone methyltransferase 1
1.99					ILMN_1230087	Psme4	proteasome (prosome, macropain) activator subunit 4
1.90					ILMN_2655005	Wnk1	WNK lysine deficient protein kinase 1
2.14					ILMN_2629591	Asah1	N-acylsphingosine amidohydrolase 1
2.09					ILMN_1215167	Ddx5	DEAD (Asp-Glu-Ala-Asp) box polypeptide 5
2.08					ILMN_2663114	Skp2	S-phase kinase-associated protein 2 (p45)
2.05					ILMN_1215013	Tshb	thyroid stimulating hormone, beta subunit
1.94					ILMN_2772274	Sfrs5	serine/arginine-rich splicing factor 5
1.97					ILMN_3132361	Ncoa4	nuclear receptor coactivator 4
2.41					ILMN_3161626	Prkag2	protein kinase, AMP-activated, gamma 2 non-catalytic subunit
2.01					ILMN_2844097	Foxn3	forkhead box N3
2.07					ILMN_2432040	Zzz3	zinc finger, ZZ domain containing 3
1.98					ILMN_3142440	Zfp386	zinc finger protein 386 (Kruppel-like)
2.24					ILMN_1236758	Wfdc2	WAP four-disulfide core domain 2
2.16					ILMN_2852380	Srp54	signal recognition particle 54A
1.97					ILMN_1239463	Snf1lk	salt inducible kinase 1
2.17					ILMN_3028837	Pcdh9	protocadherin 9
2.17					ILMN_3007428	Sox9	SRY-box containing gene 9
2.03					ILMN_1213376	Cfl1	cofilin 1, non-muscle
2.12					ILMN_2736925	Bcdin3d	BCDIN3 domain containing
2.17					ILMN_2738475	Rbm6	RNA binding motif protein 6
2.12					ILMN_1213958	Sec23a	SEC23A
2.46					ILMN_2708303	Actn3	actinin alpha 3
1.97					ILMN_2735174	Cul4a	cullin 4A
2.18					ILMN_2659151	Thbs1	thrombospondin 1
2.26					ILMN_2750669	Rngtt	RNA guanylyltransferase and 5'-phosphatase
2.33					ILMN_2972585	Pvr	poliovirus receptor
2.27					ILMN_2606693	Insig1	insulin induced gene 1
2.04					ILMN_1232851	Rab12	RAB12, member RAS oncogene family
2.21					ILMN_2446559	Tnfrsf4	tumor necrosis factor receptor superfamily, member 4
1.96					ILMN_1221266	Cugbp1	CUG triplet repeat, RNA binding protein 1
2.20					ILMN_2685569	Pus10	pseudouridylate synthase 10
2.08					ILMN_2751539	Sdcbp	syndecan binding protein

2.34					ILMN_2861176	Calr	calreticulin
2.15					ILMN_2653312	Bmpr1a	bone morphogenetic protein receptor, type 1A
2.09					ILMN_2639036	Hspd1	heat shock protein 1
2.35					ILMN_2759945	Ptpn12	protein tyrosine phosphatase, non-receptor type 12
2.27					ILMN_2898878	Pdcd4	programmed cell death 4
2.17					ILMN_2913716	H2-Ab1	histocompatibility 2, class II antigen A, beta 1
2.04					ILMN_2632942	Sumo1	SMT3 suppressor of mif two 3 homolog 1
2.08					ILMN_3160581	Pawr	PRKC, apoptosis, WT1, regulator
2.10					ILMN_2606990	Asph	aspartate-beta-hydroxylase
2.27					ILMN_1248368	Mat2a	methionine adenosyltransferase II, alpha
2.12					ILMN_2684987	Hnrnph1	heterogeneous nuclear ribonucleoprotein H1
2.40					ILMN_2593368	Mat2a	methionine adenosyltransferase II, alpha
2.30					ILMN_2659062	Add1	adducin 1
2.03					ILMN_2705578	Snx30	sorting nexin family member 30
2.06					ILMN_1232184	Ggcx	gamma-glutamyl carboxylase
2.08					ILMN_2748336	Elf2	E74-like factor 2
2.28					ILMN_2748795	Prtg	protogenin homolog
2.07					ILMN_2632940	Sumo1	SMT3 suppressor of mif two 3 homolog 1
2.09					ILMN_1234171	Ezh2	enhancer of zeste homolog 2
5.19					ILMN_1217061	Casp9	caspase 9
1.99					ILMN_2960822	BC085271	
2.25					ILMN_2645439	Bmi1	Bmi1 polycomb ring finger oncogene
2.08					ILMN_1250913	9930023K05Rik	
2.04					ILMN_1215969	Rcor1	REST corepressor 1
2.73					ILMN_2619200	Eraf	erythroid associated factor
2.11					ILMN_2499356	Xrn2	5'-3' exoribonuclease 2
2.25					ILMN_2742675	Rock1	Rho-associated coiled-coil containing protein kinase 1
2.36					ILMN_2759943	Ptpn12	protein tyrosine phosphatase, non-receptor type 12
2.31					ILMN_2747543	Actn3	actinin alpha 3
2.39					ILMN_2607675	H2-Ab1	histocompatibility 2, class II antigen A, beta 1
2.33					ILMN_1227675	Slc4a1	solute carrier family 4 (anion exchanger), member 1
2.28					ILMN_2957614	Adss	adenylosuccinate synthetase, non muscle
2.32					ILMN_2852034	Hif1a	hypoxia inducible factor 1, alpha subunit
2.23					ILMN_2671644	Cbfb	core binding factor beta
2.24					ILMN_2621086	Fli1	Friend leukemia integration 1
2.07					ILMN_1249547	Zfp292	zinc finger protein 292
5.07					ILMN_2867158	3110007F17Rik	
2.68					ILMN_2747430	Atp7a	ATPase, Cu++ transporting, alpha polypeptide
2.28					ILMN_3150990	Dach1	dachshund 1
2.28					ILMN_2727967	Cep57	centrosomal protein 57
2.33					ILMN_2733330	Rps3a	ribosomal protein S3A
2.38					ILMN_1259294	Tmem126b	transmembrane protein 126B
2.23					ILMN_2854920	Mogat2	monoacylglycerol O-acyltransferase 2
2.28					ILMN_2742626	Spin	spindlin 1
2.19					ILMN_3117714	Mex3c	mex3 homolog C
3.17					ILMN_2696696	Gypa	glycophorin A
2.34					ILMN_2815515	Zmym1	zinc finger, MYM domain containing 1
2.33					ILMN_2745513	Rhag	Rhesus blood group-associated A glycoprotein
2.33					ILMN_1213167	Eif4g2	eukaryotic translation initiation factor 4, gamma 2
2.12					ILMN_2678547	Lypla1	lysophospholipase 1
2.53					ILMN_1243615	Gramd4	GRAM domain containing 4
2.27					ILMN_2490495	Inadl	InaD-like
2.30					ILMN_2894355	Prox1	prospero-related homeobox 1
2.28					ILMN_2776034	Gal	galanin
2.53					ILMN_2631423	H2-Ab1	histocompatibility 2, class II antigen A, beta 1
2.20					ILMN_2726496	Cugbp1	CUG triplet repeat, RNA binding protein 1
2.47					ILMN_2651197	Ptpn12	protein tyrosine phosphatase, non-receptor type 12
2.33					ILMN_1255592	Sfmbt1	Scm-like with four mbt domains 1
2.29					ILMN_1237993	Nasp	nuclear autoantigenic sperm protein
2.32					ILMN_2615855	Hnrnpa2b1	heterogeneous nuclear ribonucleoprotein A2/B1
2.28					ILMN_3034098	Cnot6	CCR4-NOT transcription complex, subunit 6
2.42					ILMN_2944843	AC163646	
2.43					ILMN_2654906	Mgat3	mannoside acetylglucosaminyltransferase 3
2.32					ILMN_2653681	Mtmr2	myotubularin related protein 2
2.46					ILMN_2783997	Trim10	tripartite motif-containing 10
2.21					ILMN_1253779	Polh	polymerase (DNA directed), eta (RAD 30 related)

2.24					ILMN_2814865	Qpct	glutaminyl-peptide cyclotransferase (glutaminyl cyclase)
2.21					ILMN_2759944	Ptpn12	protein tyrosine phosphatase, non-receptor type 12
2.32					ILMN_1235689	Wdr37	WD repeat domain 37
2.78					ILMN_2752009	Mcm4	minichromosome maintenance deficient 4 homolog
2.60					ILMN_1257574	Esm1	endothelial cell-specific molecule 1
2.30					ILMN_2967455	Ppp1r13b	protein phosphatase 1, regulatory (inhibitor) subunit 13B
2.73					ILMN_1225816	Hbb-bh1	hemoglobin Z, beta-like embryonic chain
2.20					ILMN_2744228	Lipt2	lipoyl(octanoyl) transferase 2
2.26					ILMN_2958410	Stk3	serine/threonine kinase 3
2.49					ILMN_2595478	Sall4	sal-like 4
2.44					ILMN_2752010	Mcm4	minichromosome maintenance deficient 4 homolog
2.29					ILMN_2870549	Dnajc3	DnaJ (Hsp40) homolog, subfamily C, member 3
2.72					ILMN_2592779	Klhl9	kelch-like 9
2.65					ILMN_1259714	Cdk6	cyclin-dependent kinase 6
2.33					ILMN_2709681	Hdlbp	high density lipoprotein (HDL) binding protein
2.43					ILMN_2620131	Fam45a	family with sequence similarity 45, member A
2.46					ILMN_2705849	Decr2	2-4-dienoyl-Coenzyme A reductase 2, peroxisomal
2.57					ILMN_2929300	Atrx	alpha thalassemia/mental retardation syndrome X-linked
2.32					ILMN_3148398	Eprs	glutamyl-prolyl-tRNA synthetase
2.34					ILMN_1246564	Erlin2	ER lipid raft associated 2
2.38					ILMN_2705119	Btg1	B cell translocation gene 1, anti-proliferative
2.44					ILMN_2654068	Med23	mediator complex subunit 23
2.32					ILMN_1224619	St3gal1	ST3 beta-galactoside alpha-2,3-sialyltransferase 1
2.76					ILMN_2910295	Crim1	cysteine rich transmembrane BMP regulator 1
2.50					ILMN_2756578	Flrt3	fibronectin leucine rich transmembrane protein 3
2.80					ILMN_2598402	Parp8	poly (ADP-ribose) polymerase family, member 8
2.53					ILMN_1238167	Pop4	processing of precursor 4
2.35					ILMN_2675523	Slc25a30	solute carrier family 25, member 30
2.62					ILMN_1243283	matr3	matrin 3
2.47					ILMN_3027262	Gm784	fibronectin type III domain containing 3C1
2.46					ILMN_1245272	Insig1	insulin induced gene 1
2.47					ILMN_2691157	Dctn1	dynactin 1
3.05					ILMN_2634358	Msh2	mutS homolog 2
3.89					ILMN_1258136	Ccdc88a	coiled coil domain containing 88A
2.61					ILMN_2668996	Kel	Kell blood group
2.93					ILMN_2881620	Nfe2	nuclear factor, erythroid derived 2
2.70					ILMN_2617629	Mogat2	monoacylglycerol O-acyltransferase 2
2.46					ILMN_2633697	Skp2	S-phase kinase-associated protein 2 (p45)
2.62					ILMN_2719513	Slc39a8	solute carrier family 39 (metal ion transporter), member 8
2.55					ILMN_2666347	Vezt	vezatin, adherens junctions transmembrane protein
3.31					ILMN_3133817	Arhgap12	Rho GTPase activating protein 12
2.54					ILMN_1227602	Denr	density-regulated protein
2.75					ILMN_2903889	Zfp870	zinc finger protein 870
3.09					ILMN_2689230	Slc6a15	solute carrier family 6, member 15
3.06					ILMN_1233129	Zbtb22	zinc finger and BTB domain containing 22
3.24					ILMN_2744789	Cwc22	CWC22 spliceosome-associated protein homolog
3.12					ILMN_2958382	Chd1	chromodomain helicase DNA binding protein 1
3.44					ILMN_1216073	Rab3b	RAB3B, member RAS oncogene family
3.88					ILMN_2604224	Sema5a	sema domain, transmembrane domain, 5A
3.12					ILMN_2985484	Sclt1	sodium channel and clathrin linker 1
2.86					ILMN_2670662	Csde1	cold shock domain containing E1, RNA binding
3.11					ILMN_3145474	Lipt2	lipoyl(octanoyl) transferase 2
3.58					ILMN_2773540	Thap4	THAP domain containing 4
3.20					ILMN_1235423	Celsr1	cadherin, EGF LAG seven-pass G-type receptor 1
5.07					ILMN_1228040	Ptcd3	pentatricopeptide repeat domain 3
2.88					ILMN_2817151	Chchd8	coiled-coil-helix-coiled-coil-helix domain containing 8
3.18					ILMN_2647628	Ppp1cb	protein phosphatase 1, catalytic subunit, beta isoform
2.87					ILMN_2754253	Gna13	guanine nucleotide binding protein, alpha 13
3.13					ILMN_2505307	Yme1l1	YME1-like 1
3.35					ILMN_2647627	Ppp1cb	protein phosphatase 1, catalytic subunit, beta isoform
3.06					ILMN_2751492	Frag1	post-GPI attachment to proteins 2
3.47					ILMN_2600315	Ercc8	excision repaiross-complementing rodent repair deficiency, 8
3.81					ILMN_1226525	H2-Ab1	histocompatibility 2, class II antigen A, beta 1
4.36					ILMN_2992339	V2r8	vomeronasal 2, receptor, pseudogene 159
3.06					ILMN_2700166	Ccnd2	cyclin D2
3.84					ILMN_1227023	Mdm1	transformed mouse 3T3 cell double minute 1

3.75					ILMN_2477324	Trim10	tripartite motif-containing 10
3.95					ILMN_1241211	Gucy1a3	guanylate cyclase 1, soluble, alpha 3
5.09					ILMN_2604226	Sema5a	sema domain, transmembrane domain, 5A
4.75					ILMN_2435814	Trim11	tripartite motif-containing 11
3.44					ILMN_2722129	Hps1	Hermansky-Pudlak syndrome 1 homolog
3.44					ILMN_2886468	Fbxo46	F-box protein 46
3.37					ILMN_2663679	Mrpl48	mitochondrial ribosomal protein L48
4.41					ILMN_2697092	2410002O22Rik	
4.26					ILMN_3004302	Rnf41	ring finger protein 41
3.48					ILMN_2860964	Med23	mediator complex subunit 23
5.04					ILMN_2687140	B3galt6	UDP-Gal:betaGal beta 1,3-galactosyltransferase, polypeptide 6
3.58					ILMN_1216122	Mrpl48	mitochondrial ribosomal protein L48
4.58					ILMN_1249654	Sfrs16	splicing factor, arginine/serine-rich 16
6.28					ILMN_2620069	Polr1a	polymerase (RNA) I polypeptide A
3.92					ILMN_2594593	Mpp5	membrane protein, palmitoylated 5
4.95					ILMN_1232697	Zfp93	zinc finger protein 93
3.98					ILMN_1214866	Eml1	echinoderm microtubule associated protein like 1
6.61					ILMN_1246790	Chd1	chromodomain helicase DNA binding protein 1
4.92					ILMN_1214850	Pak1	p21 protein (Cdc42/Rac)-activated kinase 1
4.67					ILMN_2939138	Bicc1	bicaudal C homolog 1
5.09					ILMN_2608184	Il6st	interleukin 6 signal transducer
4.87					ILMN_1251839	Myo7a	myosin VIIA
6.29					ILMN_1243179	Sec24d	Sec24 related gene family, member D
5.54					ILMN_2838564	Tsku	tsukushin
6.47					ILMN_1250860	4732456N10Rik	
6.89					ILMN_2466926	Zfp235	zinc finger protein 235
7.48					ILMN_2594103	Rps3	ribosomal protein S3
17.07					ILMN_2753924	Mrpl35	mitochondrial ribosomal protein L35
7.24					ILMN_2921163	Rsf1	remodeling and spacing factor 1
9.24					ILMN_1238486	Ctsc	cathepsin C
8.40					ILMN_2769656	Picalm	phosphatidylinositol binding clathrin assembly protein
8.90					ILMN_2729447	9030612M13	zinc finger protein 871
10.34					ILMN_2966034	Zfp365	zinc finger protein 365
8.90					ILMN_2993661	Trim59	tripartite motif-containing 59
9.31					ILMN_2948014	AA388235	
13.01					ILMN_1217118	Enpp5	ectonucleotide pyrophosphatase/phosphodiesterase 5
9.35					ILMN_2632964	BC031748	cDNA sequence BC031748
19.44					ILMN_1235372	Hbb-b1	hemoglobin, beta adult major chain
11.86					ILMN_1223734	Atf4	activating transcription factor 4
14.25					ILMN_1238733	Ttc27	tetratricopeptide repeat domain 27
13.74					ILMN_1256643	Wdr46	WD repeat domain 46
12.47					ILMN_1238801	Arl3	ADP-ribosylation factor-like 3
13.69					ILMN_3008859	Ctsc	cathepsin C
16.26					ILMN_2508595	Zfp35	zinc finger protein 35
14.91					ILMN_2656894	Wdr46	WD repeat domain 46
21.33					ILMN_2731523	Bat5	HLA-B associated transcript 5
19.13					ILMN_2507182	Tomm22	translocase of outer mitochondrial membrane 22 homolog
23.80					ILMN_2786567	Nln	neurolysin
34.62					ILMN_3008858	Ctsc	cathepsin C
32.79					ILMN_2818206	Mettl14	methyltransferase like 14
-15.23					ILMN_2729513	Hbb-b2	hemoglobin, beta adult major chain 2
-6.14					ILMN_2729826	Sst	somatostatin
-4.62					ILMN_3000679	Folr1	folate receptor 1 (adult)
-4.57					ILMN_2946653	Klk1b22	kallikrein 1-related peptidase b22
-4.91					ILMN_2707541	Folr1	folate receptor 1 (adult)
-3.74					ILMN_3162394	Ppp2r5c	protein phosphatase 2, regulatory subunit B, gamma isoform
-3.54					ILMN_1248563	Car11	carbonic anhydrase 11
-3.19					ILMN_2815889	Grwd1	glutamate-rich WD repeat containing 1
-4.24					ILMN_2734090	Vstm2l	V-set and transmembrane domain containing 2-like
-3.83					ILMN_2699052	Nrn1	neuritin 1
-3.60					ILMN_2939277	Sncg	synuclein, gamma
-3.24					ILMN_1226016	Scx	scleraxis
-3.05					ILMN_2598916	Actc1	actin, alpha, cardiac muscle 1
-4.03					ILMN_2598478	Sncg	synuclein, gamma
-2.77					ILMN_2441534	Rsph1	radial spoke head 1 homolog
-4.65					ILMN_1225704	Stmn3	stathmin-like 3

-2.83					ILMN_2797061	Actn2	actinin alpha 2
-2.54					ILMN_2614494	Fah	fumarylacetoacetate hydrolase
-3.36					ILMN_2738825	Acta1	actin, alpha 1, skeletal muscle
-2.93					ILMN_2918875	Sln	sarcolipin
-3.20					ILMN_2840975	Gls2	glutaminase 2
-2.79					ILMN_1223735	Myh8	myosin, heavy polypeptide 8, skeletal muscle, perinatal
-2.84					ILMN_2769777	Msc	musculin
-2.40					ILMN_1251909	Vars2	valyl-tRNA synthetase 2, mitochondrial
-3.06					ILMN_1251725	Ly6g6c	lymphocyte antigen 6 complex, locus G6C
-2.48					ILMN_2977331	Mylpf	myosin light chain, phosphorylatable, fast skeletal muscle
-2.49					ILMN_2944610	Islr2	immunoglobulin superfamily containing leucine-rich repeat 2
-2.42					ILMN_3104118	AW548124	protein kinase domain containing, cytoplasmic
-3.61					ILMN_2938820	Nefm	neurofilament, medium polypeptide
-2.50					ILMN_2610744	Myl4	myosin, light polypeptide 4
-2.31					ILMN_2624153	Hes5	hairy and enhancer of split 5
-2.93					ILMN_3065852	Kif5a	kinesin family member 5A
-2.40					ILMN_2657822	Stat2	signal transducer and activator of transcription 2
-2.18					ILMN_1221007	Prkd3	protein kinase D3
-2.35					ILMN_2460136	Tnnt1	troponin T1, skeletal, slow
-2.59					ILMN_2463181	Tnc	tenascin C
-2.24					ILMN_1240303	Lmo1	LIM domain only 1
-3.15					ILMN_2836494	Ina	internexin neuronal intermediate filament protein, alpha
-2.86					ILMN_3128792	Ttn	titin
-2.66					ILMN_2882658	Tnnc2	troponin C2, fast
-2.16					ILMN_2917386	Gstp2	glutathione S-transferase, pi 2
-2.44					ILMN_2503052	Tnnc1	troponin C, cardiac/slow skeletal
-2.79					ILMN_2922321	Slc17a6	solute carrier family 17, member 6
-2.30					ILMN_1212692	Mapk13	mitogen-activated protein kinase 13
-3.38					ILMN_2988818	2810416G20Rik	
-2.04					ILMN_1252105	Ankrd24	ankyrin repeat domain 24
-3.19					ILMN_3043469	Ret	ret proto-oncogene
-2.28					ILMN_1218223	Pvalb	parvalbumin
-2.39					ILMN_1226042	Cplx1	complexin 1
-2.34					ILMN_2696182	Actl6b	actin-like 6B
-2.14					ILMN_2737903	Fgd2	FYVE, RhoGEF and PH domain containing 2
-2.21					ILMN_2631903	1700008P20Rik	
-2.74					ILMN_3102035	Elavl4	LAV (embryonic lethal, abnormal vision, Drosophila)-like 4
-2.38					ILMN_3031009	Capzb	capping protein (actin filament) muscle Z-line, beta
-2.20					ILMN_2463180	Tnc	tenascin C
-2.21					ILMN_2828896	Pxdn	peroxidasin homolog
-2.09					ILMN_2846373	Cript	cysteine-rich PDZ-binding protein
-3.23					ILMN_2846865	Actb	actin, beta
-2.39					ILMN_1253414	Hes5	hairy and enhancer of split 5
-2.38					ILMN_1250114	Mast1	microtubule associated serine/threonine kinase 1
-2.11					ILMN_2677595	Ncapg2	non-SMC condensin II complex, subunit G2
-2.17					ILMN_2985428	Actl6b	actin-like 6B
-2.39					ILMN_2762640	Gls2	glutaminase 2
-2.46					ILMN_1253304	Stmn2	stathmin-like 2
-2.46					ILMN_2878542	Myl1	myosin, light polypeptide 1
-2.45					ILMN_2680398	Zc3h12d	zinc finger CCCH type containing 12D
-3.61					ILMN_2827729	Calb2	calbindin 2
-2.77					ILMN_2805839	Sez6l	seizure related 6 homolog like
-2.25					ILMN_2604070	Celsr3	cadherin, EGF LAG seven-pass G-type receptor 3
-2.22					ILMN_2616841	Sgip1	SH3-domain GRB2-like (endophilin) interacting protein 1
-2.15					ILMN_2955452	Mapk13	mitogen-activated protein kinase 13
-2.42					ILMN_3040710	Rtn1	reticulon 1
-1.96					ILMN_2813724	Bai2	brain-specific angiogenesis inhibitor 2
-2.35					ILMN_1229379	Syt13	synaptotagmin XIII
-2.14					ILMN_2693913	Onecut2	one cut domain, family member 2
-2.09					ILMN_2630182	Syp	synaptophysin
-3.48					ILMN_2696126	Smg8	smg-8 homolog, nonsense mediated mRNA decay factor
-2.48					ILMN_2962361	Rufy3	RUN and FYVE domain containing 3
-3.20					ILMN_2588055	Actb	actin, beta
-2.37					ILMN_2798973	Myt1	myelin transcription factor 1
-2.14					ILMN_1235388	Igsf21	immunoglobin superfamily, member 21
-2.98					ILMN_2980815	Pcdh21	protocadherin 21

-2.01	ILMN_2931623	Dcx	doublecortin
-1.93	ILMN_1239196	1700088E04Rik	
-2.15	ILMN_1239547	Gcnt1	glucosaminyl (N-acetyl) transferase 1, core 2
-2.54	ILMN_1235230	Pdlim3	PDZ and LIM domain 3
-1.88	ILMN_1228479	Kremen	kringle containing transmembrane protein 1
-2.41	ILMN_2765454	Svop	SV2 related protein
-1.98	ILMN_2863674	Myog	myogenin
-2.01	ILMN_2976441	Blk	B lymphoid kinase
-2.28	ILMN_2593225	Prx	periaxin
-2.15	ILMN_2646606	St8sia6	ST8 alpha-N-acetyl-neuraminide alpha-2,8-sialyltransferase 6
-2.06	ILMN_1244675	Crmp1	collapsin response mediator protein 1
-1.89	ILMN_1255254	Serinc2	serine incorporator 2
-2.00	ILMN_1248788	Lypd2	Ly6/Plaur domain containing 2
-1.92	ILMN_1251874	Pscd2	cytohesin 2
-1.99	ILMN_2749595	Inf2	inverted formin, FH2 and WH2 domain containing
-2.39	ILMN_1228206	Dcx	doublecortin
-1.98	ILMN_2950622	Arhgdig	Rho GDP dissociation inhibitor (GDI) gamma
-1.95	ILMN_2598946	Bai2	brain-specific angiogenesis inhibitor 2
-2.90	ILMN_2725531	1700028K03Rik	
-2.21	ILMN_2789650	Csrp3	cysteine and glycine-rich protein 3
-2.25	ILMN_1233130	Ablim3	actin binding LIM protein family, member 3
-2.15	ILMN_3162042	Rhox10	reproductive homeobox 10
-2.05	ILMN_2890145	Fam163a	family with sequence similarity 163, member A
-2.09	ILMN_2609813	Chi3l1	chitinase 3-like 1
-1.89	ILMN_2667352	Glo1	glyoxalase 1
-1.94	ILMN_2805144	Capn8	calpain 8
-1.89	ILMN_2703267	Nes	nestin
-2.39	ILMN_2823947	Ccdc110	coiled-coil domain containing 110
-2.34	ILMN_1247343	Lmcd1	LIM and cysteine-rich domains 1
-2.08	ILMN_1235133	Syn1	synapsin I

-2.0 0 2.0

Supplementary Table S1

GO term	Genes
**disease**	
glaucoma	*Actb, Atp7a, Bmpr1a, Calb2, Calr, Casp9, Cbfb, Ccnd2, Cd200, Chi3l1, Col18a1, Dcx, Ddah2, Ednrb, Epas1, Esm1, Gal, Glo1, Hif1a, Hspd1, Mtmr2, Myog, Nefm, Nes, Nrn1, Pvalb, Pvr, Pxdn, Ret, Rock1, Rock2, Sncg, Sox2, Srsf5, Sst, Syp, Thbs1, Tnc, Tyrp1, Vamp8*
cataract	*Actb, Ahsp, Angpt1, Asph, Atf4, Atp7a, Bmpr1a, Capzb, Casp9, Cdk6, Chm, Col18a1, Cxcr7, Dcx, Fah, Glo1, Gypa, Hba-x, Hbb-b1, Hbb-b2, Hif1a, Hps1, Klc1, Lgsn, Morf4l1, Mtmr2, Nefm, Prox1, Pxdn, Rhag, Sec23a, Sgk3, Slc25a30, Slc4a1, Sox2, Sst, Thbs1, Tnc, Tyrp1*
eye neoplasm	*Actb, Angpt1, Bmi1, Calb2, Casp9, Ccne2, Cdh11, Cdk6, Col18a1, Dcx, Ednrb, Epas1, Ezh2, Fli1, Hif1a, Hnrnpf, Hnrnph1, Hspd1, Ivns1abp, Mcm6, Msh2, Mtap1b, Myog, Nes, Pak1, Pdcl, Pvalb, Pvr, Pvrl2, Ret, Rhag, Sall4, Sdcbp, Skp2, Sncg, Sox2, Sox9, Sst, Syp, Thbs1, Tnc, Tnfrsf4, Tyrp1*
optic nerve disease	*Abat, Actb, Atf4, Calb2, Calr, Casp9, Ccnd2, Chm, Crmp1, Dctn1, Dcx, Ednrb, Epha3, H2-Ab1, Hif1a, Hspd1, Il6st, Mtap1b, Myo7a, Nes, Pawr, Pvalb, Ret, Rock2, Slc17a6, Sncg, Sox2, Sst, Syn1, Syp, Tnc, Tshb, Ttn, Tyrp1*
**tissue**	
retina	*Abat, Arl3, Atrx, B3galt4, Calb2, Casp9, Cdhr1, Chm, Col18a1, Cplx1, Dach1, Dcx, Epas1, Epha3, Folr1, Hes5, Hif1a, Lmcd1, Mdm1, Melk, Mpp5, Mtap1b, Myo7a, Nefm, Nes, Ppp2r5c, Prox1, Pvalb, Slc17a6, Sncg, Sox2, Sst, St8sia1, Stmn3, Syn1, Syp, Thbs1*
optic disc	*Ednrb, Hif1a, Hspd1, Rock1, Tnc*
cornea	*Actb, Nes, Rock2, Thbs1, Tnc,*
optic nerve	*Casp9, Ednrb, Fyn, Hif1a, Ina, Mtap1b, Nefm, Nes, Sema5a, Sncg, Tnc*
